# The reversion of DNA methylation-induced miRNA silence via biomimetic nanoparticles-mediated gene delivery for efficient lung adenocarcinoma therapy

**DOI:** 10.1186/s12943-022-01651-4

**Published:** 2022-09-28

**Authors:** Lu Liang, Huiyu Cen, Jionghua Huang, Aiping Qin, Wenyan Xu, Siran Wang, Zhijun Chen, Lin Tan, Qiqi Zhang, Xiyong Yu, Xin Yang, Lingmin Zhang

**Affiliations:** 1 Guangzhou Municipal and Guangdong Provincial Key Laboratory of Molecular Target & Clinical Pharmacology, the NMPA and State Key Laboratory of Respiratory Disease, School of Pharmaceutical Sciences and the Fifth Affiliated Hospital, Guangzhou Medical University, Guangzhou, 511436 China; 2 Department of Cardiovascular Disease, The Third Affiliated Hospital, Guangzhou Medical University, Guangzhou, 510150 China; 3 Department of Preventive Dentistry, Guangdong Engineering Research Center of Oral Restoration and Reconstruction, Guangzhou Key Laboratory of Basic and Applied Research of Oral Regenerative Medicine, Affiliated Stomatology Hospital of Guangzhou Medical University, 510182 Guangzhou, China; 4 Department of Medical Imaging, Affiliated Cancer Hospital & Institute of Guangzhou Medical University, Guangzhou, 510095 China

**Keywords:** Biomimetic nanoparticles, Lung adenocarcinoma, MicroRNA, MMP2, DNA methylation

## Abstract

**Background:**

Lung cancer is one of the fatal cancers worldwide, and over 60% of patients are lung adenocarcinoma (LUAD). Our clinical data demonstrated that DNA methylation of the promoter region of miR-126-3p was upregulated, which led to the decreased expression of miR-126-3p in 67 cases of lung cancer tissues, implying that miR-126-3p acted as a tumor suppressor. Transduction of miR-126-3p is a potential therapeutic strategy for treating LUAD, yet the physiological environment and properties of miRNA challenge current transduction approaches.

**Methods:**

We evaluated the expression of miR-126-3p in 67 pairs of lung cancer tissues and the corresponding adjacent non-tumorous tissues by Reverse transcription-quantitative polymerase chain reaction (RT-qPCR). The relationship between the overall survival of lung cancer patients and miR-126-3p was analyzed by the Cancer Genome Atlas cohort database (Oncolnc, http://www.oncolnc.org). We analyzed DNA methylation Methylation-specific PCR (MSP) analysis. To determine whether ADAM9 is the direct target of miR-126-3p, we performed the 3′-UTR luciferase reporter assay. The protein levels in the cells or tissues were evaluated with western blotting (WB) analysis. The biodistribution of nanoparticles were monitored by in vivo tracking system.

**Results:**

We describe the development of novel stealth and matrix metalloproteinase 2 (MMP2)-activated biomimetic nanoparticles, which are constructed using MMP2-responsive peptides to bind the miR-126-3p (known as MAIN), and further camouflaged with red blood cell (RBC) membranes (hence named REMAIN). REMAIN was able to effectively transduce miRNA into lung cancer cells and release them via MMP2 responsiveness. Additionally, REMAIN possessed the advantages of the natural RBC membrane, including extended circulation time, lower toxicity, better biocompatibility, and immune escape. Moreover, in vitro and in vivo results demonstrated that REMAIN effectively induced apoptosis of lung cancer cells and inhibited LUAD development and progression by targeting ADAM9.

**Conclusion:**

The novel style of stealth and MMP2-activated biomimetic nanoparticles show great potential in miRNA delivery.

**Supplementary Information:**

The online version contains supplementary material available at 10.1186/s12943-022-01651-4.

## Introduction

Lung cancer is one of the fatal cancers worldwide, and over 60% of patients are lung adenocarcinoma (LUAD) [1]. Conventional treatments, such as chemotherapy, radiotherapy, and surgical resection, show limited therapeutic benefit and significant side effects for the patients. Previous studies have reported that the microRNA (miRNA), miR-126-3p, is frequently downregulated in many types of human cancers due to the DNA methylation of CpG islands in miRNA promoter regions, which changes the tumor-suppressive properties [2–4]. Although miR-126-3p has potential efficacy in treating cancers, the physiochemical properties limit its applications in this field. For example, the strongly negative charges of the miRNA hamper internalization by the cell membrane, which is also negatively charged. In addition, rapid enzymatic digestion in the physiological environment hinders the systemic delivery of naked miRNAs to the desired sites in the body [5, 6]. These challenges necessitate the development of a durable and effective miRNA delivery system.

Numerous vehicles, including viral and nonviral vectors, have been developed to carry the miRNA; however, viral vectors show several disadvantages, such as limited packaging, inflammatory/immunogenic responses, and difficulty in manufacturing at a large-scale [7]. The application of nonviral vectors for gene delivery has recently attracted more attention because they can be designed conveniently, have low cytotoxicity, and show intelligent responsiveness [8–10]. The nonviral vectors include liposomes, polymers, and organic/inorganic nanoparticles [11–16]. To transduce the nucleic acid into cancer cells, the vectors must overcome three trafficking barriers: (1) escape immunity, (2) be taken up effectively by cancer cells, and (3) protect and release the therapeutic nucleic acids [17, 18]. The currently available nonviral vectors are also challenged by practical applications, such as short circulation lifetime, low environmental responsiveness, and complex preparation procedures.

The biomimetic nanoparticles encapsulated with cell membranes exhibit remarkable advantages by combining the properties of natural cell membranes with those of artificial core materials [19]. Cell membrane-encapsulated nanoparticles possess many benefits, including extended circulation, specific targeting, and immune escape [20, 21]. Red blood cells (RBCs), the oxygen transporters in our body, have a lifespan of approximately four months and project many surface markers, which have been considered as a bionic prototype to develop the drug delivery systems based on the RBCs-derived membranes (RBCM). The extended circulation lifetime of RBCs-coated nanoparticles is mainly mediated by various membrane proteins, such as CD47, a self-recognized protein. The inner nanoparticles were endowed with well encapsulation and self-recognition after the coating with RBCM, which prevents immune cells’ phagocytosis and improves the circulation lifetime.

Cancer cells often overexpress the matrix metalloproteinase 2 (MMP2), in ranges of 10 ~ 1000-fold higher than normal cells, which can degrade the MMP substrates including extracellular matrix (ECM) and synthesized biomacromolecules, providing a great opportunity to design the enzyme-responsive nanostructures [22]. MMP2 and other effectors regulate reactions in the invasion-metastasis cascade [23]. The peptide sequence Pro-Leu-Gly-Leu-Ala-Gly (PLGLAG) can be specifically recognized and cleaved by MMP2. Previous work indicated that this MMP2-cleavable peptide could be used to construct MMP2-responsive nanoparticles, which disintegrate in the presence of abundant MMP2. However, these kinds of nanoparticles have not been reported to the delivery of gene medicines for lung cancer yet. We consider that the MMP2-responsive peptide can be used to construct the environment-responsive nanoparticles that can improve the release of miRNA in the LUAD cells.

Herein, we report the development of stealth and MMP2-activated biomimetic nanoparticles to deliver the miR-126-3p for LUAD therapy. First, the cationic peptide was synthesized with six arginine residues at both ends of the MMP2-cleavable peptide PLGLAG (6R-PLGLAG-6R). We used 6R-PLGLAG-6R to combine miR-126-3p by electrostatic adsorption (MMP2 stimulated peptide/miRNA-126-3p, MAIN), which was further camouflaged with RBCM (Red blood cell membrane/MMP2 stimulated peptide/miRNA-126, REMAIN). We supposed that the MMP2-stimulated peptide could bind miRNA effectively. The camouflage with RBCM endowed the nanoparticles with a "stealth" function, allowing for the improvement in circulation lifetime, immune escape, and biosafety. After REMAIN was taken up by the lung cancer cells, miR-126-3p is released in the presence of abundant MMP2 in the lung cancer cells, effectively inducing apoptosis of the cancer cell (Scheme 1). Furthermore, we also investigated the mechanism of tumor inhibition induced by miR-126-3p. Our work explored and developed a novel strategy with great promise for LUAD therapy.

**Figure:**
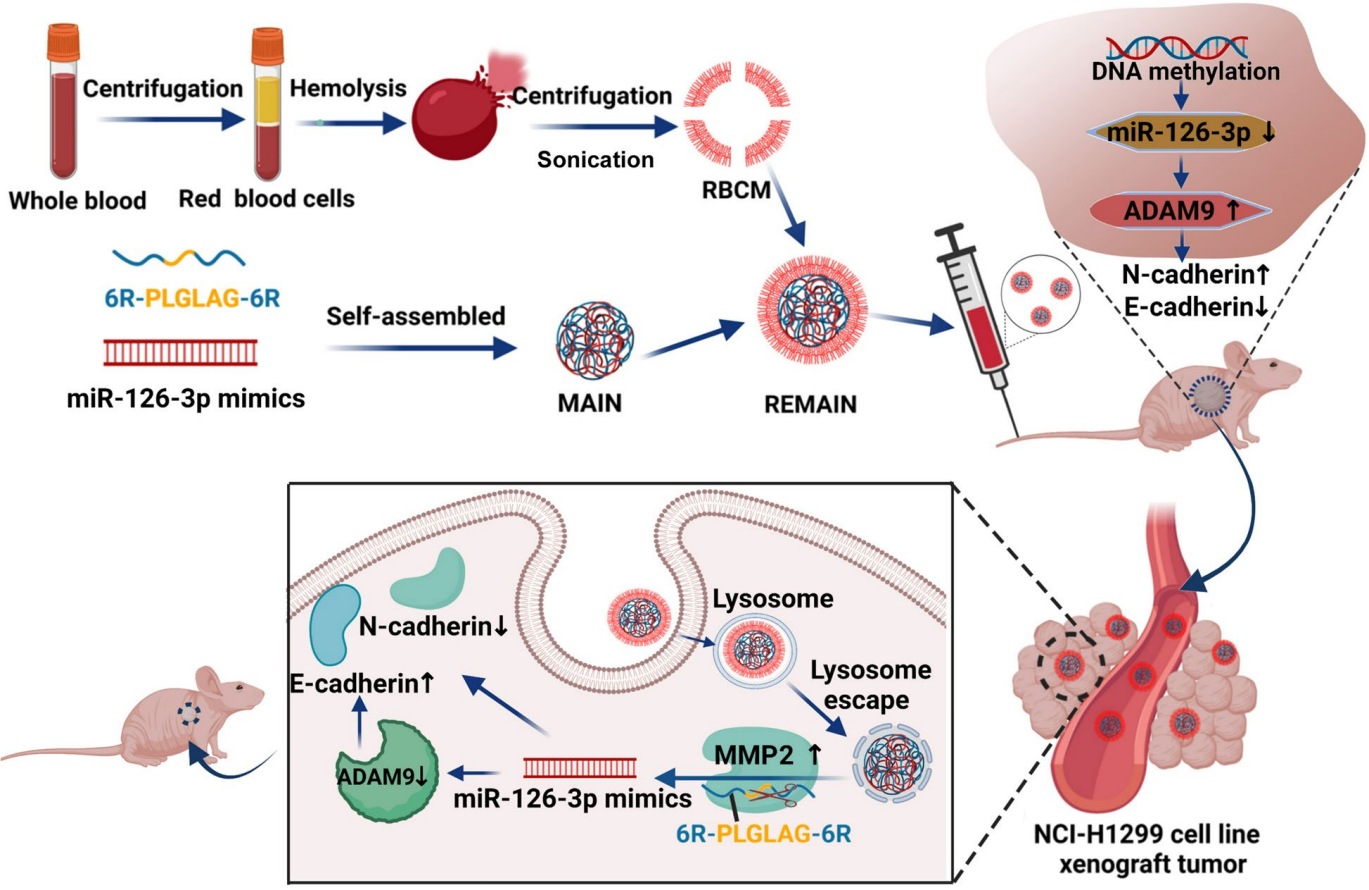
**Scheme 1**

## Materials and methods

### Patients and clinical samples

All experimental protocols were approved by the Ethics Committee of the Affiliated Cancer Hospital of Guangzhou Medical University (202,108,004). Informed consent was received from all participants before enrollment. 67 pairs of tumor and adjacent normal tissues were obtained from surgeries at the Affiliated Cancer Hospital of Guangzhou Medical University (Guangzhou, Guangdong, China).

### Cell culture

Human lung carcinoma cell lines H460, NCI-H1299, A549, and the normal human lung epithelial cell line BEAS-2B were obtained from ATCC (American Type Culture Collection, USA). hUV-MSCs and HUVECs were purchased from ScienCell Research Laboratories. The cells were cultured in DMEM supplemented with 10% FBS, 100 μg/mL streptomycin, and 100 U/mL penicillin in an incubator containing 5% CO_2_ at 37 °C.

### Preparation of MAIN and REMAIN 

The positively charged polypeptide 6R-PLGLAG-6R and negatively charged miR-126-3p self-assemble to form nanoparticles. 6R-PLGLAG-6R (GL Biochem Ltd, Shanghai, China) and miR-126-3p (Genepharma, Shanghai, China) were dissolved in RNase free water to prepare at concentrations of 10 mg/mL and 1 mg/mL, respectively. To screen the appropriate ratio of 6R-PLGLAG-6R to miR-126-3p, the mass ratio (w/w) of miR-126-3p to 6R-PLGLAG-6R was set as 1:5, 1:10, 1:20, 1:30, 1:40, 1:50, 1:60, and 1:90, respectively. Next, the mixture was vortexed immediately for 1 min and incubated for 15 min to obtain MAIN.

Red blood cell membranes were collected using the red blood cell lysis buffer according to the manufacturer's instructions (Beyotime Biotechnology, China). The red blood cell membranes were used to wrap MAIN following the mass ratio (w/w) of MAIN to RBCM at 1:5, 1:10, 1:20, and 1:30, respectively. The mixtures were sonicated for 5 min, and then incubated at room temperature for 15 min to obtain REMAIN. We also prepared the RBCM-encapsulated nanoparticles with negative miRNA control (REMAIN-NC).

### Characterization of MAIN and REMAIN

The suspension of MAIN or REMAIN was prepared in deionized water. The Zeta Sizer (Malvern, UK) was used to measure the mean particle size, zeta potential, and PDI of the MAIN and REMAIN in different proportions of nanoparticles. The morphology of the MAIN or REMAIN was analyzed by TEM (JEOL, Japan). The protein spectrum of REMAIN was analyzed by Coomassie blue staining.

The loading efficiency of miR-126-3p was determined by detecting the unbound miR-126-3p in the supernatant. Next, the content of unbound miR-126-3p in the supernatant was measured by agarose gel electrophoresis.

### Cell viability assay

Cell viability was evaluated by a CCK-8 kit (Beyotime Biotechnology, China) according to the manufacturer's protocol. The ratio of cell viability was calculated as A _treated_/A _cont rol_ × 100%.

### Luciferase reporter assay

The experimental protocol was followed as previously described [24]. The DNA fragment containing the wild-type (WT) or mutant (MT) 3ʹ-UTR of ADAM9 was synthesized and inserted into the pGL3 vector to generate WT ADAM9-3ʹ-UTR or MT ADAM9-3ʹ-UTR reporter.

### Colony formation assay

The colony formation assay followed the standard protocol [25]. We counted the number of colonies, and the cloning efficiency was calculated as follows: cloning efficiency (%) = (the number of cell colonies / the number of seeded cells) × 100.

### Western blotting (WB) assay

An equal amount of total protein was run on 12.5% SDS-PAGE, transferred to PVDF membranes (250 mA for 2 h), and probed with primary antibodies. The primary antibodies include anti-MMP2 (1:1000, 72 kDa, Sigma-Aldrich), anti-N-cadherin (1:1000, 140 kDa, ImmunoWay Biotechnology), anti-E-cadherin (1:1000, 135 kDa, ImmunoWay Biotechnology), anti-Vimentin (1:1000, 57 kDa, ImmunoWay Biotechnology), anti-ADAM9 (1:1000, 72 kDa, Affinity Biosciences), anti-Snail (1:1000, 29 kDa, ImmunoWay Biotechnology), anti-DNMT1 (1:1000, 183 kDa, Abcam), anti-CD47 (1:1000, 52 kDa, Abcam), and anti-GAPDH (1:1000, 37 kDa, Sigma-Aldrich). The protein bands of interest were captured after the secondary antibodies linked with peroxidase were bound to primary antibodies [26].

### Wound healing and Transwell invasion assays

Transwell invasion and wound healing assays were performed following the previously published approaches [27] to evaluate the effect of PBS, Lipo3000/miR-126-3p, REMAIN, MAIN, REMAIN-NC, free miR-126-3p, or si-ADAM9 on cell invasion and migration, respectively.

### Serum nuclease protection assay

Free miR-126-3p, MAIN, or REMAIN (miR-126-3p equivalent to 150 nM) were incubated in DMEM medium containing 10% FBS at 37 ℃ from 15 min to 6 h. Agarose gel electrophoresis was used to evaluate the residual miR-126-3p content and visualized by the Amersham Imager 600 system (GE Healthcare Life Sciences, USA).

### In vitro miR-126-3p release study

First, REMAIN containing FITC-miR-126-3p (Guangzhou RiboBio, China) was prepared to analyze the miR-126-3p release. The miR-126-3p release experiments were performed with or without MMP2 enzyme. An aliquot (1 mL) of the dialysate was taken at different time points (1–72 h), and then 1 mL of fresh dialysate was added. The miR-126-3p (Guangzhou RiboBio, China) in the collected dialysate was measured using a NanoDrop One (Thermo Fisher Scientific, U.S.A.).

### Cellular uptake study

NCI-H1299 cells were seeded into confocal dishes at 1 × 10^5^ cells per well and culture in DMEM complete medium. After 24h, the cells were incubated in Opti-MEM medium containing REMAIN with different concentrations of Cy5-miR-126-3p (Guangzhou RiboBio, China) equivalent to 50 nM, 100 nM, 150 nM, and 200 nM for 9 h, or REMAIN with Cy5-miR-126-3p equivalent to 150 nM incubated for different time points (1–12 h). Meanwhile, NCI-H1299 cells were incubated in an Opti-MEM medium containing REMAIN, MAIN, or free Cy5-miR-126-3p for 9 h. Then, DAPI and FITC-Phalloidin were used to stain the cells. Cell imaging was performed by confocal laser scanning microscopy (CLSM, Zeiss 880, Germany) to qualitatively display the cellular uptake of nanoparticles. To quantitatively analyze the cellular uptake, the cells were collected and suspended in PBS and analyzed by flow cytometry (FACS, Amnis Corporation, Seattle, WA).

To evaluate the MMP2 level in the culture medium, NCI-H1299 cells were seeded into 24-well plates at 1 × 10^5^ cells per well and culture in DMEM complete medium. After 24 h, the medium was removed and replaced with 1 mL Opti-MEM. The culture medium was obtained at the time points of 0, 1, 3, 6, 9, and 12 h, respectively. To analyze quantitatively the MMP2 level, the culture medium was analyzed by MMP2 ELISA Kit (Abcam plc., USA).

### The study of endocytosis mechanism

To study the endocytosis mechanism of the NCI-H1299 cells, the cells were pretreated to 4 ℃ environment, 2.5 μg/mL chlorpromazine (CPZ), 50 μg/mL genistein (GEN), and 200 μg/mL amiloride (AMI) for 2 h, and then treated with REMAIN for 9 h. After 9 h, the cells were analyzed with CLSM and FACS.

### The transfection efficiency in vitro

NCI-H1299 cells were treated with PBS, Lipo3000/miR-126-3p, MAIN, REMAIN-NC or free miR-126-3p (miR-126-3p equivalent to 150 nM), respectively. After 48 h, the expression levels of miR-126-3p were evaluated by RT-qPCR assay [28].

### Reverse transcription-quantitative polymerase chain reaction (RT-qPCR) assay

Total RNAs were extracted using the Trizol reagent, and 500 ng of total RNAs were used for cDNA synthesis by *Evo M-MLV RT* Kit according to the manufacturer’s protocol. Then 1 μL of cDNA and SYBR Green were used for qPCR detecting. Primers used for RT-qPCR are listed in Table S1. Differences among target expressions were quantitatively analyzed using U6 and GAPDH as internal reference genes.

### Methylation analysis MSP assay

The primers specific for the methylated (M) and unmethylated (U) miRNA‑126-3p promoter are shown in Table S2. The protocol for MSP was as follows: 95 °C for 5 min; followed by 40 cycles at 95 °C for 30 s, 55 °C for 30 s, and 72 °C for 30 s; and extension at 72 °C for 10 min. PCR products were separated on 2% agarose gels.

### Lysosomal escape assay

The NCI-H1299 cells were treated with REMAIN containing 150 nM Cy5-miR-126-3p (Guangzhou RiboBio, China) for 1 and 9 h, respectively. The cells were stained with LysoTracker Green (Thermo Fisher Scientific Inc., USA) for 45 min and fixed with 4% paraformaldehyde at 37 ℃ for 15 min. The confocal laser scanning microscope was used to detect and display the distribution of nanoparticles at different time points.

### Tube formation assay

The HUVEC cells treated with PBS, Lipo3000/miR-126-3p, REMAIN, MAIN, REMAIN-NC, or free miR-126-3p (miR-126-3p equivalent to 150 nM) were seeded onto Matrigel-coated 96-well plates. The cells were incubated at 37 °C with 5% CO_2_ for 6 h and observed under a microscope. The number of tubes was analyzed by Image 6.0.

### Live/Dead assay

The NCI-NCI-H1299 cells with a density of 1 × 10^5^ per well were seeded into confocal dishes. PBS, Lipo3000/miR-126-3p, REMAIN, MAIN, REMAIN-NC or free miR-126-3p (miR-126-3p equivalent to 150 nM) were separately transfected into NCI-H1299 cells. The cells were stained using the Live/Dead Kit (Thermo Fisher Scientific Inc., USA) and observed by a confocal laser scanning microscope.

### In vivo tracking study

All animal studies were approved by the Institutional Animal Care and Use Committee of Guangzhou Medical University and performed in compliance with NIH guidelines for the care and use of laboratory animals (GY2022-027). Six-week-old male BALB/c nude mice were obtained from Beijing HFK Bioscience (Beijing, China; license number SCXK 2019–0010).

To investigate the targetability of the nanoparticles in vivo, we prepared REMAIN (containing miR-126-3p, 20 μg per mouse), MAIN (containing miR-126-3p, 20 μg per mouse) and free miR-126-3p (20 μg per mouse) (miR-126-3p labeled with Cy5) for tracking study. Male BALB/c nude mice were implanted with 2 × 10^6^ NCI-H1299 cells at the right flank regions. After the tumors grew to about 200 mm^3^, the prepared nanoparticles were injected intravenously through the tail vein of BALB/C nude mice bearing NCI-H1299 tumor. At 1–48 h after injection, images were taken using the IVIS Lumina imaging system, and the whole blood was collected to measure the concentration of Cy5-miR-126-3p. After in vivo tracking, the tumors and tissues of BALB/C nude mice were collected and subjected to ex vivo imaging.

### The antitumor effects of REMAIN

The male BALB/C nude mice with subcutaneous NCI-H1299 tumor xenografts were randomly divided into 6 groups (*n* = 5). The mice in each group were injected through the tail vein with normal saline, REMAIN-NC, free miR-126-3p (20 μg per mouse), MAIN (containing miR-126-3p, 20 μg per mouse), REMAIN (containing miR-126-3p, 20 μg per mouse), and Gefitinib (20 mg/kg), respectively. The body weight and tumor volume were recorded throughout the experiments. At the end of the experiment, the mice were euthanized immediately, and the main organs (hearts, lungs, livers, spleens, and kidneys) and tumors were excised. The collected tissues and tumors were used for H&E staining and TUNEL analysis.

### Statistical analysis

The data were analyzed using GraphPad Prism 5.0 software. All data were expressed as mean ± standard deviation (SD). Intergroup differences were analyzed using the Student’s *t*-test when two groups were compared, or one-way ANOVA was used when multiple groups were compared. *P* < 0.05 was considered statistically significant.

## Results

### miR-126-3p expression is silenced by hypermethylation of its promoter in lung cancer cells

**Fig. 1:**
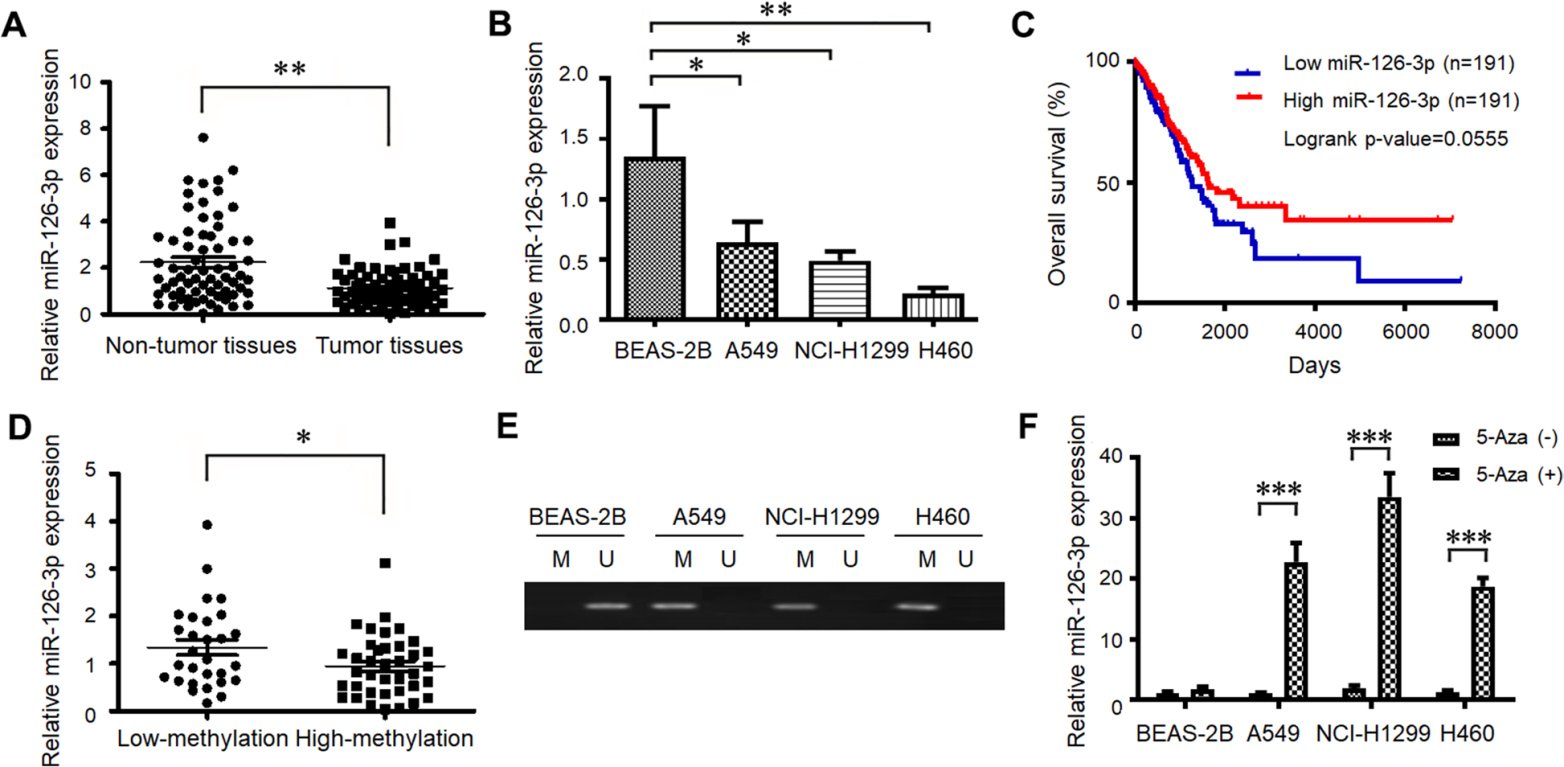
miR-126-3p expression is silenced by hypermethylation of its promoter in lung cancer cells. **A** The miR-126-3p levels were measured by RT-qPCR in 67 pairs of lung adenocarcinoma tissues and adjacent non-tumor tissues. **B** RT-qPCR detected the miR-126-3p expression levels in BEAS-2B, A549, NCI-H1299, and H460 cells. **C** The overall survival of lung cancer patients with low or high miR-126-3p expression. **D** The relative expression levels of methylation of miR-126-3p were detected in tumor samples and adjacent lung samples by qRT-PCR. **E** MSP for methylation or demethylation of the miRNA-126-3p gene promoter in different LUAD and BEAS-2B cells. **F** The relative expression levels of miR-126-3p in different LUAD and BEAS-2B cells (treated with or without 5-Aza-dC for 24 h) were measured by RT-qPCR. *, *P* < 0.05; **, *P* < 0.01; ***, *P* < 0.001

To investigate the role of miR-126-3p in LUAD, we first evaluated the expression of miR-126-3p in 67 pairs of lung cancer tissues and the corresponding adjacent non-tumorous tissues by Reverse transcription-quantitative polymerase chain reaction (RT-qPCR). miR-126-3p expression levels were much lower in tumor tissues than in adjacent non-tumor tissues (Fig. 1A). Additionally, the expression levels of miR-126-3p were examined in three types of LUAD cell lines (A549, NCI-H1299, and H460) and normal human bronchial epithelial cells (BEAS-2B) as a control. Consistent with the LUAD tissue results, the levels of miR-126-3p expression in all the lung cancer cells were less than that in BEAS-2B cells (Fig. 1B). The relationship between the overall survival of lung cancer patients and miR-126-3p was analyzed by the Cancer Genome Atlas cohort database (Oncolnc, http://www.oncolnc.org). These results demonstrated that lower miR-126-3p levels were associated with a worse overall survival rate in lung cancer patients (Fig. 1C). Taken together, the downregulated expression of miR-126-3p in both LUAD tissues and cells suggested that miR-126-3p might act as a tumor suppressor in LUAD development.

To understand the association between miR-126-3p methylation and clinicopathologic parameters, we detected the miR-126-3p methylation levels in 67 LUAD patients. We found the rate of miR-126-3p methylation levels was higher in tumor tissues than in adjacent tissues. As shown in Table S3, in the 67 LUAD tissues, 38 cases exhibited methylation, and 29 were unmethylated. However, all the adjacent normal tissues were nonmethylated (0/67). The 67 clinical cases were classified as high-methylation (38/67, 56.7%) and low-methylation (29/67, 43.3%). Higher levels of miRNA-126-3p expression were significantly observed in the low-methylation group compared to the high-methylation one (Fig. 1D). Furthermore, in 28 patients with lymph node metastasis, over half of the samples (22/28) were methylated, indicating a significant difference (*P* = 0.0022). The rates of miR-126-3p methylation in the well and moderately differentiated groups and poorly differentiated groups were 67.6% and 43.3%, respectively, suggesting a significant difference (*P* = 0.0465). The results indicated that miR-126-3p methylation levels were not significantly associated with age or gender. Consequently, we found that methylation of the miR-126-3p promoter was strongly correlated with lymph node metastasis and pathological differentiation in LUAD.

Because the inactivation of tumor suppressor genes is closely associated with epigenetic silencing, we further investigated whether the expression of miR-126-3p is regulated by methylation in LUAD cells. Methylation-specific PCR (MSP) analysis indicated that DNA methylation in miR-126-3p occurred in A549, NCI-H1299, and H460 cells, while BEAS-2B cells were unmethylated (Fig. 1E). LUAD cells treated with 5-aza-2’-deoxycytidine (5-Aza-dC), a demethylating agent, increased miR-126-3p levels compared to cells without treatment (Fig. 1F). To better understand the correlation between DNA Methyltransferase 1 (DNMT1) and miR-126-3p, we silenced DNMT1 by siRNA (Figure S1A). Our results demonstrated that silencing DNMT1 upregulated miR-126-3p expression in A549 and NCI-H1299 cells (Figure S1B). Thus, the inhibition of DNMT1 led to the increase of miR-126-3p, indicating a negative correlation between DNMT1 and miR-126-3p. As shown in Figure S1C and D, the knockdown of DNMT1 suppressed the mRNA and protein expressions of ADAM9 in A549 and NCI-H1299 cells. The data demonstrated that epigenetic factors could affect the expression of miR-126-3p, and DNA methylation might be an important mechanism for the function of miR-126-3p in lung adenocarcinoma progression.

### miR-126-3p acts as a negative regulator of ADAM9 in LUAD

**Fig. 2:**
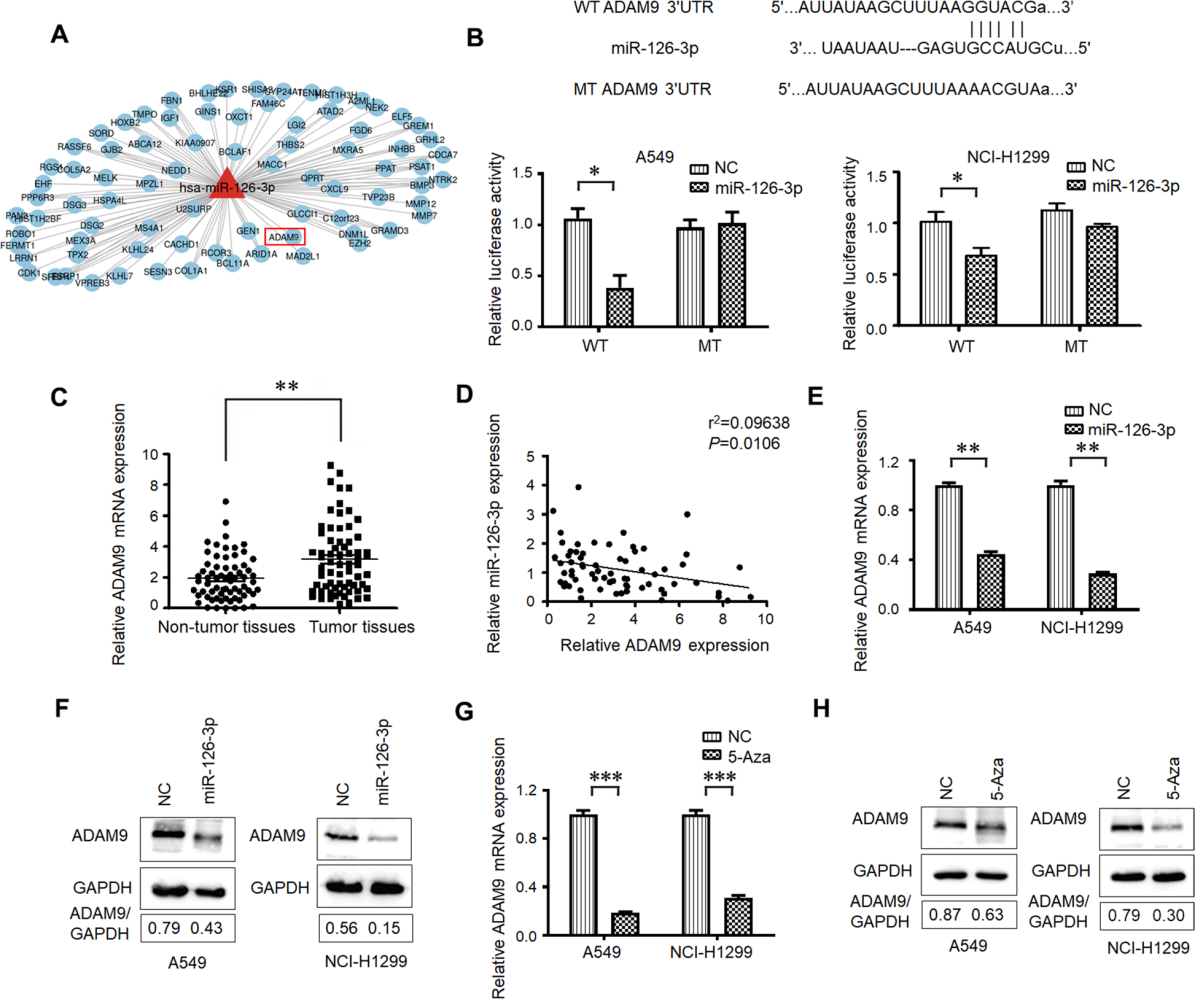
miR-126-3p is a negative regulator of ADAM9 in LUAD. **A** The target genes of miR-126-3p screened by mirDB database. **B** Diagram of putative miR-126-3p binding sequence in ADAM9 3′-UTR and its mutant in luciferase reporter assay. A luciferase reporter assay was performed to evaluate luciferase activity in A549 and NCI-H1299 cells. **C** ADAM9 mRNA levels were evaluated in lung adenocarcinoma tissues and adjacent lung tissues. **D** The correlation of miR-126-3p expression to mRNA expression in lung adenocarcinoma tissues. The mRNA (**E**) and protein (**F**) levels of ADAM9 in miR-126-3p mimic-transfected A549 and NCI-H1299 cells. The mRNA (G) and protein (H) levels of ADAM9 in A549 and NCI-H1299 cells treatment with 5-Aza, respectively. *, *P* < 0.05; **, *P* < 0.01; ***, *P* < 0.001

Known interaction networks between the differentially expressed miR-126-3p and the target genes were searched in the mirDB database (http://www.mirdb.org/), and ADAM9 was identified (Fig. 2A). To determine whether ADAM9 is the direct target of miR-126-3p, we performed the 3′-UTR luciferase reporter assay by the use of pGL3 reporter constructs containing wild-type or mutated ADAM9 3′-UTR fragments. The overexpression of miR-126-3p substantially reduced the luciferase activity of the ADAM9-WT reporter in A549 and NCI-H1299 cells (Fig. 2B). However, these effects were not observed in the mutated ADAM9-MT groups, suggesting that ADAM9 is the target gene of miR-126-3p. Moreover, we also measured the expression of ADAM9 mRNA in 67 freshly collected lung cancer tissues and adjacent non-tumorous tissues. Compared with the matched non-tumorous tissues, lung cancer tissues exhibited higher expression levels of ADAM9 mRNA (Fig. 2C). A significant inverse correlation was confirmed between the levels of miR-126-3p and ADAM9 mRNA expression (Fig. 2D). Subsequently, we examined the mRNA and protein levels of ADAM9. Transfection with the miR-126-3p mimic decreased ADAM9 expression in both A549 and NCI-H1299 cells (Fig. 2E and F). Furthermore, we also studied the effect of miR-126-3p inhibition on ADAM9 expression. After the LUAD cells were treated with miR-126-3p inhibitor, the ADAM9 mRNA levels were upregulated significantly (Figure S2). To investigate the effects of DNA demethylating agent on the mRNA and protein levels of ADAM9, we treated A549 and NCI-H1299 cells with 5 μM 5-Aza-dC for 48 h. After 5-Aza-dC treatment, the expression of ADAM9 was suppressed, suggesting its transcription is regulated by promoter-specific DNA methylation (Fig. 2G and H). The mentioned results confirmed that miR-126-3p acted as a negative regulator of ADAM9 in LUAD.

### The preparation and characterization of REMAIN

As demonstrated above, miR-126-3p is a negative regulator of ADAM9, a marker related to poor prognosis [29]. Although the commercial reagents, such as Lipo3000, showed effective transduction efficiency in vitro, the complex physiological environment compromised their application in vivo. To ensure the effective transduction of miR-126-3p, we developed a versatile biomimetic nanostructure to deliver miR-126-3p. First, we designed a series of peptides containing MMP2-recognizable sequences PLGLAG with 2, 4, and 6 arginine residues at both ends of these sequences (corresponding to 2R-PLGLAG-2R, 4R-PLGLAG-4R, and 6R-PLGLAG-6R, respectively), which were used to combine with miR-126-3p. Notably, 6R-PLGLAG-6R was most effective in binding with the miR-126-3p (named MAIN), which formed a smaller particle at each given weight ratio (Figure S3A). The ratio of 6R-PLGLAG-6R to miR-126-3p at 30:1 (w/w) showed an expected size of ~ 100 nm. 6R-PLGLAG-6R induced a much higher miR-126-3p expression in NCI-H1299 cells than 2R-PLGLAG-2R or treatment 4R-PLGLAG-4R. The increased arginine means the increase of aminos or iminos, which increases the charge density in the neural conditions, and improved the compact of negative-charged miR-126-3p. The miR-126-3p level was similar to the transfection by the commercial agent Lipo3000 (Figure S3B). Thus, we used 6R-PLGLAG-6R to bind miR-126-3p for further studies. We analyzed the properties of MAIN. The weight ratios of 6R-PLGLAG-6R to miR-126-3p were 5, 10, 20, 30, 40, 50, 60, and 90. Our results indicated that 6R-PLGLAG-6R to miR-126-3p weight ratios > 30 formed outstanding nanoparticles. The size of these nanoparticles ranged from 100 to 500 nm. The polydispersity index (PDI) is less than 0.5, and the zeta potential is ≈ -2 mV (Figure S4A and B). We analyzed the loading efficiency with 6R-PLGLAG-6R by detecting the unbound miR-126-3p with the weight ratio (w/w) of 6R-PLGLAG-6R to miR-126-3p from 5:1 to 90:1, respectively. The results demonstrated that the encapsulation rate was approximately 97% at the ratio of 30:1 and reached 99% over 50:1 (Figure S4C). Based on our results, an obvious high loading efficiency of 6R-PLGLAG-6R to miR-126-3p was observed.

To evaluate the safety of 6R-PLGLAG-6R, we examined the cell viability in the presence of 6R-PLGLAG-6R using the Cell Counting Kit-8 assay in vitro. We observed no obvious changes in cell viability when cells were treated with either 6R-PLGLAG-6R or the combination with the negative miRNA control (6R-PLGLAG-6R/miR-NC). More than 80% of cells remained viable at the weight ratio of 6R-PLGLAG-6R/miR-NC at 90/1 (Figure S4D). These results suggested that 6R-PLGLAG-6R showed negligible toxicity in vitro.

To improve the surface functions, we used RBCM to camouflage MAIN (named REMAIN) and investigated the conditions for the formulation of 6R-PLGLAG-6R/ miR-126-3p (MAIN) and RBCM. The fresh whole blood was obtained from the BALB/c nude mice, and red blood cells were extracted. The intracellular contents were removed using a hypotonic condition, yielding RBC ghosts. After coating MAIN with RBCM at the ratio of 1 to 30, the weight ratio of RBCM to the MAIN at 10 was chosen (size≈150 nm; PDI = 0.25; zeta potential≈ − 30 mV) (Figure S5A and B). We determined the optimized weight ratio of RBCM/6R-PLGLAG-6R/miR-126-3p (REMAIN) is 300/30/1. WB analysis indicated CD47 as the marker of RBCM was retained after the coating, confirming the successful camouflage with RBCM (Figure S5C). We also evaluated whether the process to coat RBCM on MAIN caused the loss of miR-126-3p. After the miR-126-3p was dissociated from MAIN or REMAIN, the amount of miR-126-3p in both nanoparticles was similar (Figure S5D), implying the coating process would not cause the loss of miR-126-3p significantly.

**Fig. 3:**
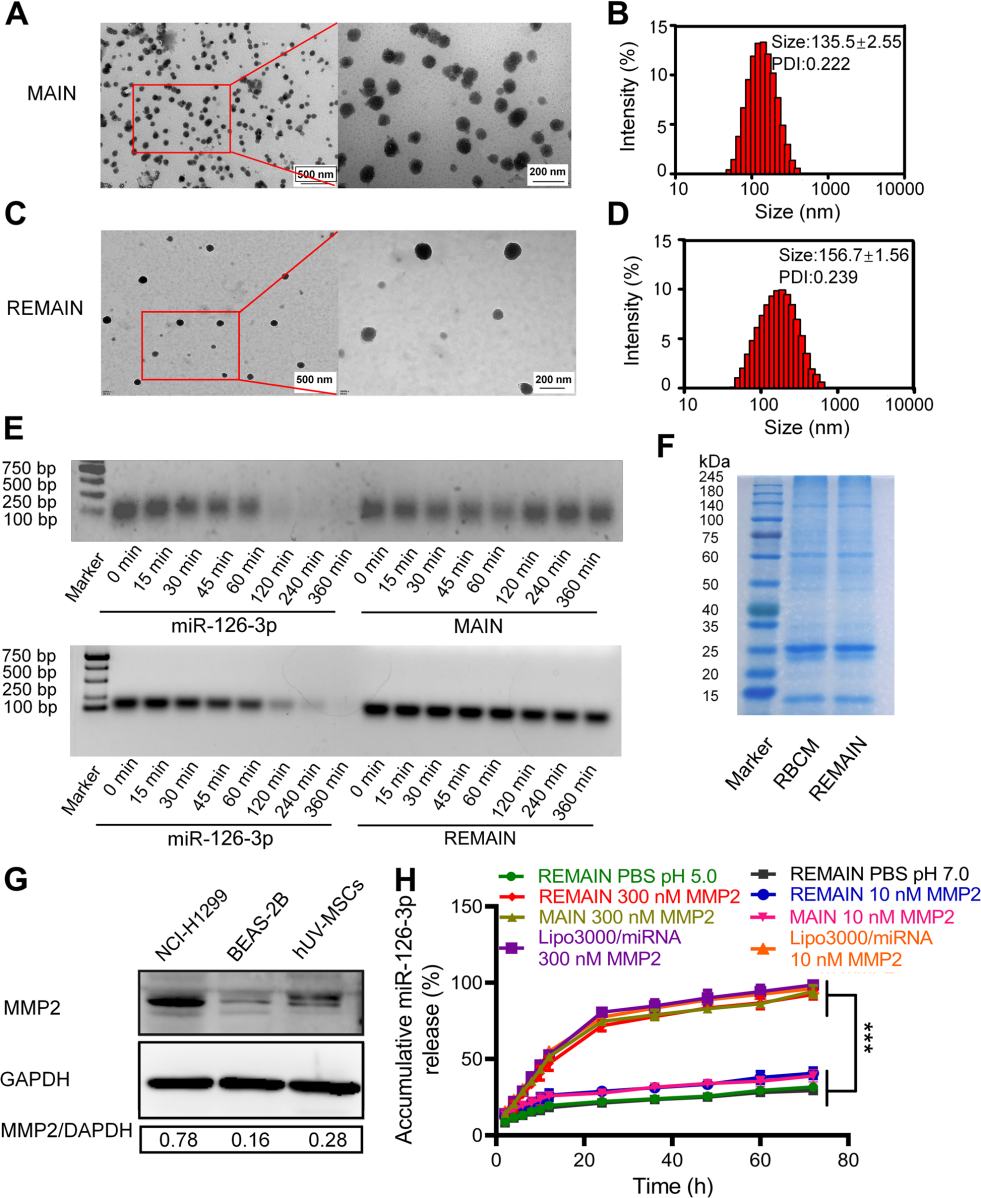
The properties of RBCM coated nanoparticles. **A** TEM image analysis and (**B**) the size distribution of MAIN. **C** TEM analysis and (**D**) the size distribution of REMAIN. **E** Nuclease protection assay of miR-126-3p. **F** SDS-PAGE analysis of REMAIN. **G** WB analysis of MMP2 levels. **H** The cumulative release of miR-126-3p in different conditions

Transmission electron microscope (TEM) and dynamic light scattering (DLS) analysis revealed that MAIN was nearly spherical with a diameter of ~ 135 nm (Fig. 3A and B). After camouflaging with RBCM, the resulting REMAIN was also spherical but with a ~ 20 nm increase in size (Fig. 3C and D). The nuclease protection assay was used to evaluate the ability of MAIN or REMAIN to protect miR-126-3p from nuclease digestion. In the miR-126-3p group, no visible bands were observed after 120 min, indicating the free miR-126-3p was digested by nucleases. However, the presence of all the bands confirmed that MAIN or REMAIN prevented the nuclease digestion of miR-126-3p (Fig. 3E). To verify the RBCM proteins on REMAIN, sodium dodecyl sulfate–polyacrylamide gel electrophoresis (SDS-PAGE) gel indicated that REMAIN retained most of the proteins from RBCM (Fig. 3F). Western blotting (WB) analysis revealed that the MMP2 levels were significantly higher in NCI-H1299 cells than in the normal cell lines, including human lung epithelial cells (BEAS-2B) and human umbilical cord mesenchymal stem cells (hUV-MSCs) (Fig. 3G). These results indicated that a high concentration of MMP2 appeared in the lung cancer cells. The drug release profiles demonstrated that REMAIN showed a slow release of miR-126-3p, with less than 30% release within 72 h incubation in the PBS (pH 5.0 and pH 7.0). At the low MMP2 environment (10 nM), both MAIN and REMAIN showed less than 30% release of miR-126-3p within 72 h. However, the miR-126-3p release from MAIN or REMAIN reached about 60% after 12 h, sharply increasing to about 75% at 24 h in the MMP2-containing medium (300 nM) (Fig. 3H). Interestingly, we found that Lipo3000 showed no significant difference with low or high MMP2, implying that the release of miR-126-3p were not dependent on MMP2. We evaluated the stability of the nanoparticles. Firstly, MAIN or REMAIN was suspended in the PBS (pH = 7.0) for 3 days. DLS analysis indicated there was more than 100 nm increase in the size of MAIN within 3 days. In contrast, REMAIN showed an increase of size ~ 50 nm in the same conditions, implying that the camouflage with RBCM improved the stability of nanoparticles (Figure S6A). Furthermore, we also evaluated the size and PDI in different medium with different FBS, and found that the RBCM camouflage endowed the nanoparticles with excellent stability with minor changes in both size and PDI (Figure S6B). The CCK-8 assay indicated that after coating with RBCM, RBCM/6R-PLGLAG-6R/miRNA-NC (REMAIN-NC) demonstrated no obvious toxicity in NCI-H1299 cells (Figure S7).

We successfully prepared MMP2-activated biomimetic nanoparticles, which were used to carry miRNA efficiently. The miRNA-loaded nanoparticles, REMAIN, were ~ 150 nm and prevented the miRNA from nuclease degradation. REMAIN also showed an MMP2-stimulated property, inducing an improved release of miRNA in the presence of MMP2.

### Evaluation of cellular uptake

The cellular uptake of REMAIN was examined by CLSM and flow cytometry (FACS). We optimized the transfection condition by adjusting the dose and transfection time. To track the cellular uptake of the nanoparticles, the fluorescent dye Cyanine 5 (Cy5) was used to label miR-126-3p (Cy5-miR-126-3p). Both CLSM and FACS analysis indicated that the incubation for 9 h reached a maximum transfection efficiency of approximately 100%, and the extension to 12 h did not show significant differences (Figure S8A). Additionally, the treatment with REMAIN (miR-126-3p equivalent to 150 nM) showed nearly 100% of cells were Cy5 positive with maximum fluorescence intensity (Figure S8B). Thus, REMAIN at a concentration of 150 nM miR-126-3p and transfection for 9 h were used in subsequent experiments.

**Fig. 4:**
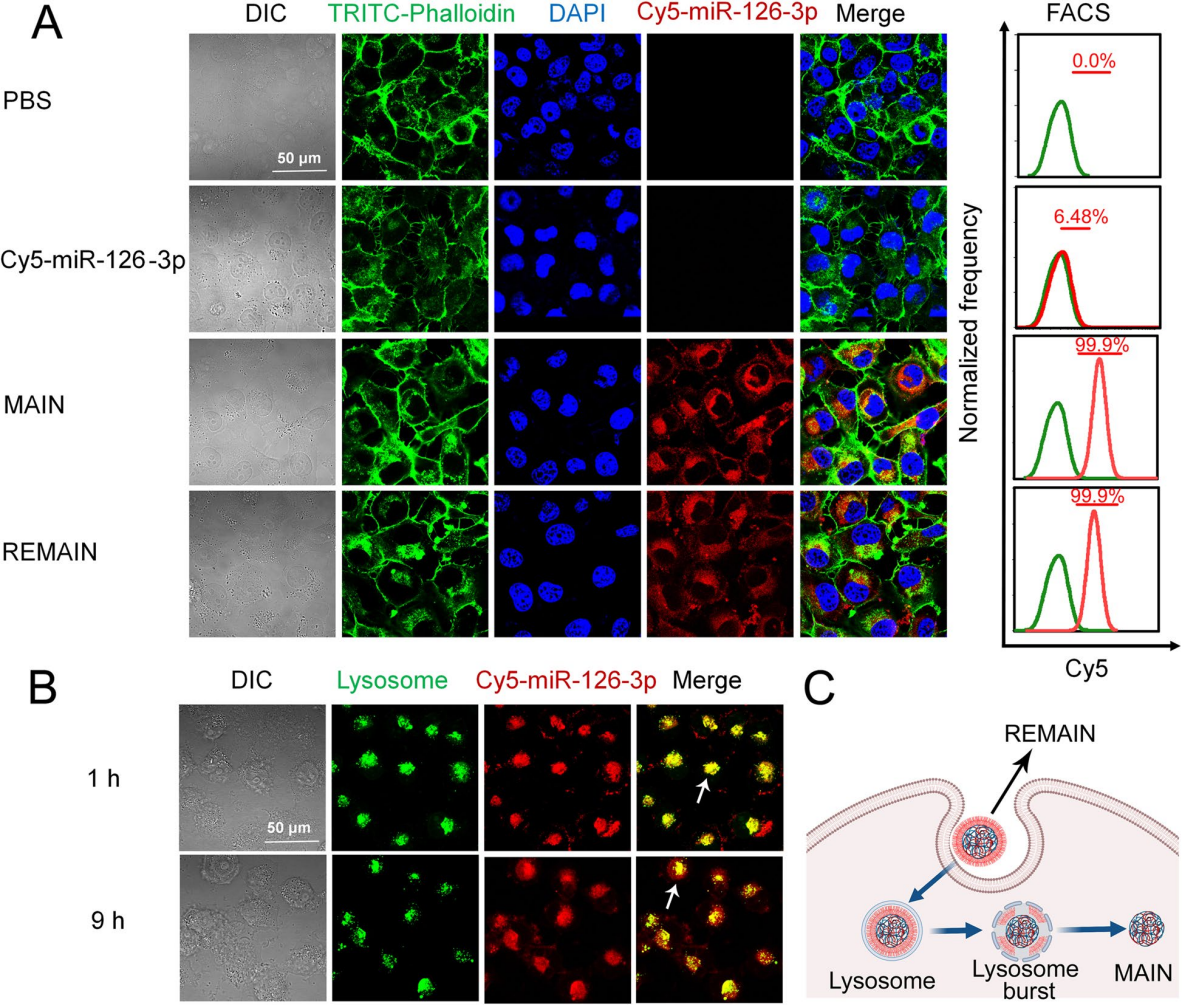
The cellular uptake in different conditions. **A** The cellular uptake of different formulations containing 150 nM miR-126-3p. **B** The lysosome escape of Cy5-miR-126-3p. **C** Scheme illustration of lysosome escape. NCI-H1299 cells were incubated with REMAIN for 1 and 9 h, respectively. Excitation/Emission: FITC-Phalloidin (488 nm/525 nm); Cy5 (633 nm/670 nm); DAPI (358 nm/461 nm)

To gain insights into the cellular uptake of different formulations, we evaluated the uptake of naked miR-126-3p, MAIN, and REMAIN by CLSM. Strong fluorescence was observed in NCI-H1299 cells after incubation with MAIN and REMAIN, indicating that MAIN and REMAIN can effectively facilitate the uptake of miR-126-3p (Fig. 4A). However, almost no red fluorescence was present in cells after incubation with naked miR-126-3p (Fig. 4A). To exclude the interference induced by the extracellular MMP2, we detected the MMP2 level in the culture medium after incubation within 12 h, and found that the MMP2 was less than 1 pM within the time (Figure S9), which confirmed that the low MMP2 would not affect the cellular uptake by disintegrating the nanoparticles.

We also studied the endocytosis mechanism. In the present study, clathrin, caveolin, or Na^+^/H^+^ exchange-mediated endocytosis was inhibited by chlorpromazine (CPZ), genistein (GEN), or amiloride (AMI), respectively. The temperature was also a potential factor to affect the cellular uptake. We found that the cellular uptake of REMAIN was reduced by ~ 95% after the pretreating the cells at 4 ℃, which was evidenced that the endocytosis process was energy dependent. The pretreatment with AMI did not affect the cellular uptake of REMAIN in NCI-H1299 cells, implying that the Na^+^/H^+^ exchange showed little effect on the uptake of REMAIN. Chlorpromazine treatment reduced the REMAIN uptake by 15% in NCI-H1299 cells, suggesting a possible involvement of the clathrin-mediated pathway. Significantly, GEN reduced the cellular uptake of the REMAIN by 40% in NCI-H1299 cells, implying that the caveolin-mediated pathway played the major role in endocytosis (Figure S10 and Figure S11). The data indicated that the cellular uptake of RBCM-camouflaged nanoparticles was energy dependent by caveolin-mediated pathway.

We also evaluated lysosome escape, which plays a crucial role in releasing loaded cargo to the cytoplasm. The lysosomes were labeled with lysotracker (green fluorescence), and miR-126-3p was labeled by Cy5 (red fluorescence). After incubation for 1 h, a large amount of REMAIN was captured by the lysosomes, as represented by the yellow fluorescence, which indicated the merger of green and red fluorescence as indicated by the white arrow. However, after 9 h, most green fluorescence separated from the red fluorescence, indicating miR-126-3p escaped from lysosomes as indicated by the white arrow (Fig. 4B and C). The cationic peptide, 6R-PLGLAG-6R might play important role in this process, which was protonated in the acidic lysosomes and led to the increase of the osmotic pressure, resulting in the lysosome burst and release of miRNA [30]. The efficient cellular uptake and lysosomal escape demonstrated that REMAIN possessed great potential to deliver miRNA into cells and improved their release from lysosomes, which ensured the therapeutic effect of miRNA on lung cancer.

### Antitumor effects of nanoparticles on LUAD cells in vitro

**Fig. 5:**
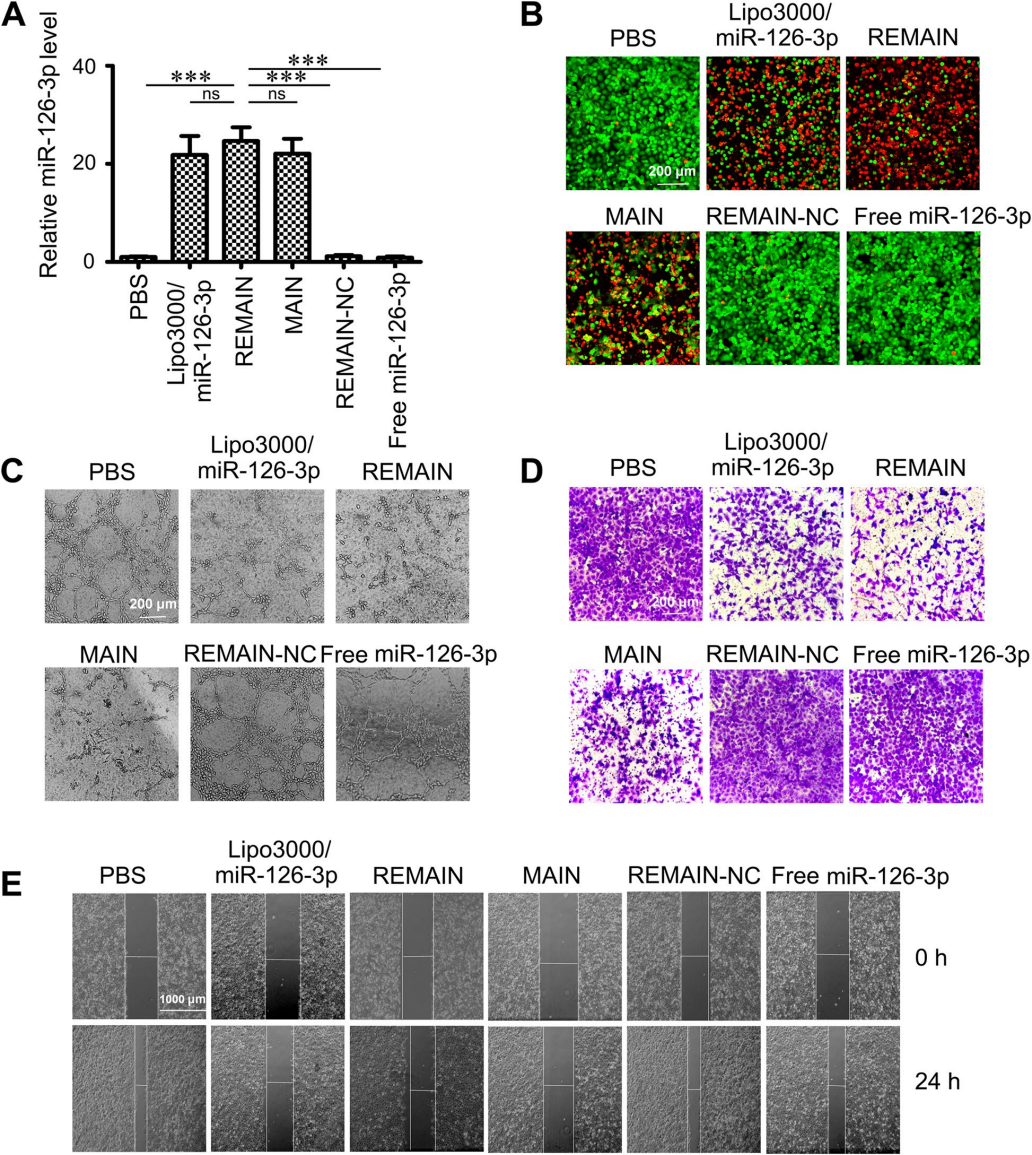
The effect of different formulations of nanoparticles on NCI-H1299 cells in vitro. **A** miR-126-3p levels, (**B**) Live/dead staining, (**C**) Tube formation of HUVECs, (**D**) Cell invasion and (**E**) Cell migration was analyzed after treatment with PBS, Lipo3000/miR-126-3p, REMAIN, MAIN, REMAIN-NC or free miR-126-3p, respectively. *, *P* < 0.05; **, *P* < 0.01; ***, *P* < 0.001

To examine the biological activities induced by different nanoparticles, we detected the expression levels of miR-126-3p by RT-qPCR in NCI-H1299 cells. As shown in Fig. 5A, the miR-126-3p levels were significantly upregulated in the cells transfected with Lipo3000/miR-126-3p, REMAIN, or MAIN, compared with the PBS group. The miR-126-3p levels in the cells treated with REMAIN and MAIN were increased by 26-fold and 23-fold, respectively, compared with non-transfected cells. Our results demonstrated REMAIN and MAIN possess considerable transfection efficiency compared with the transfection reagent Lipo3000. We evaluated the inhibition of cancer cells with a Live/Dead kit, in which Calcein was used as the live indicator with green fluorescence and propidium iodide (PI) as the dead one with red fluorescence). Lipo3000/miR-126-3p, MAIN, and REMAIN showed significant cancer cell inhibition. Approximately 70% cells were PI-positive (Fig. 5B). However, the control groups, such as REMAIN-NC, free miR-126-3p, or PBS, showed no significant cell inhibition. We also tested the effect of REMAIN and MAIN on cell viability by the Cell Counting Kit-8 (CCK-8) assay in vitro. Both REMAIN and MAIN induced a cancer cell inhibition of approximately 80%. Similar inhibition of proliferation was observed in cells transfected with miR-126-3p using Lipo3000 (Figure S12A).

It has been reported that miRNA-126-3p is essential for vascular development by maintaining vascular integrity and promoting angiogenesis [31, 32]. To further validate the anti-angiogenic effect of MAIN and REMAIN, we performed a network formation assay, and found that the untreated REMAIN-NC and free miR-126-3p treated human umbilical vein endothelial cells (HUVECs) formed stable tube-like structures (Fig. 5C). However, the number of tube-like structures decreased in Lipo3000/miR-126-3p, MAIN, and REMAIN-treated groups (Fig. 5C and S12B). Subsequently, we analyzed the role of REMAIN and MAIN in cell migration and invasion of lung cancer cells by wound healing assay and transwell assay. The invaded cells covered almost the entire lower surface of the control group. Similar results were observed when the cells were treated with REMAIN-NC and free miR-126-3p. Lipo3000/miR-126-3p, MAIN, and REMAIN significantly inhibited cell invasion with coverage rates at approximately 60% on the lower surface (Fig. 5D and S12C). The relative migration rate was calculated by the migration distance normalized to control groups. As indicated in Fig. 5E and S12D, untreated, REMAIN-NC, and free miR-126-3p treated cells exhibited strong migration healing ability. Lipo3000/miR-126-3p, MAIN, and REMAIN strongly inhibited (approximately 70%) the migration of NCI-H1299 cells. These results demonstrated that REMAIN shows effective cancer cell inhibition and anti-angiogenic effects in vitro.

### The mechanism of LUAD inhibition by REMAIN

**Fig. 6:**
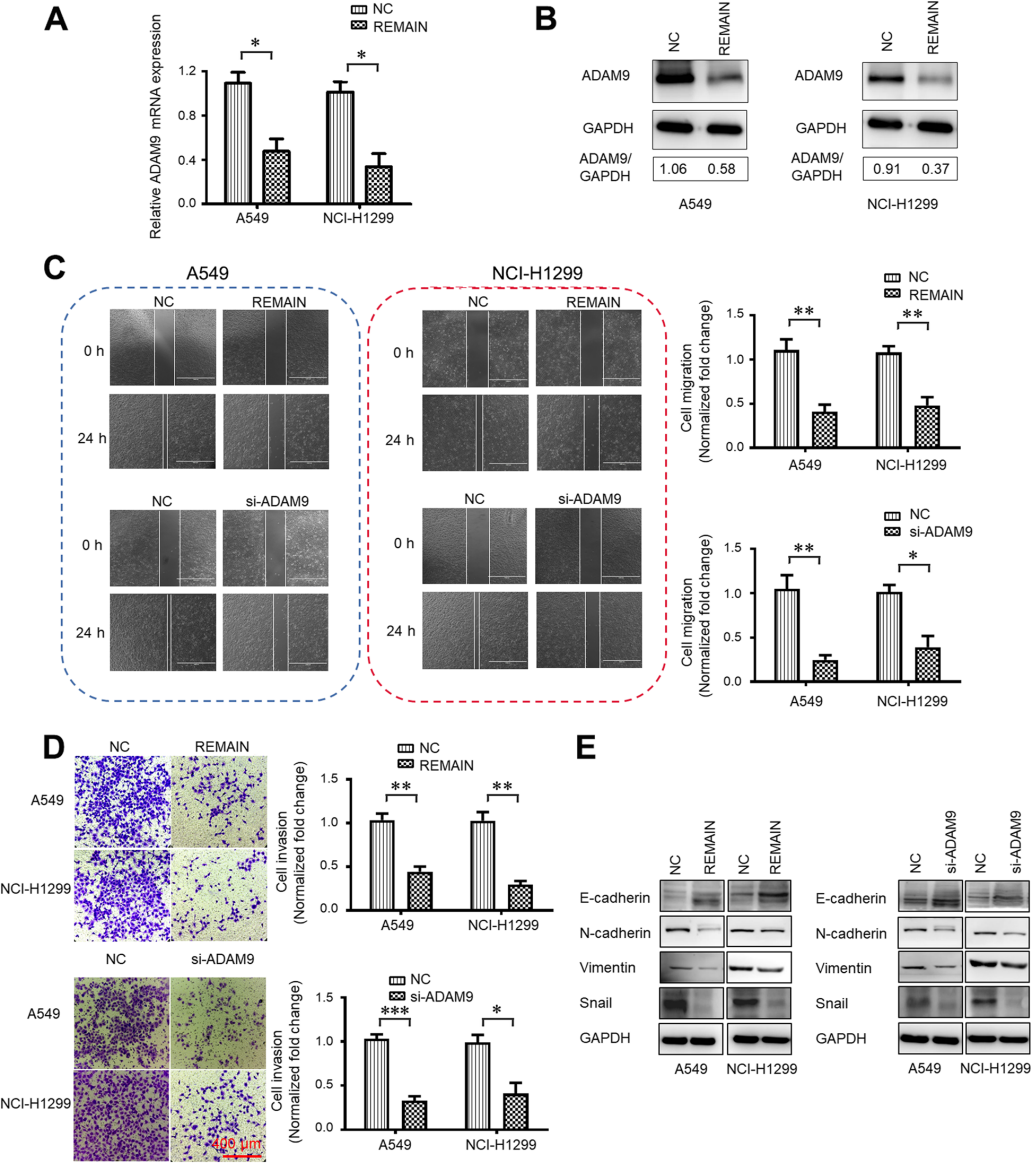
The inhibition of LUAD cell migration, invasion, and endothelial-to-mesenchymal transition. A549 and NCI-H1299 cells were treated with REMAIN or si-ADAM9 for 48 h. The mRNA (A) and protein (B) levels of ADAM9 in A549 and NCI-H1299 cells treatment with REMAIN, respectively. (C) Wound healing assays measured cell migration. (D) Transwell assays measured cell invasion. (E) Western blot assays determined the expression of EMT markers E-cadherin, N-cadherin, and Vimentin. *, *P* < 0.05; **, *P* < 0.01; ***, *P* < 0.001

We explored the mechanism of LUAD inhibition by REMAIN. The mRNA and protein levels of ADAM9 were evaluated in A549 and NCI-H1299 cells induced by REMAIN. RT-qPCR analysis indicated that ADAM9 mRNA levels were significantly decreased to approximately 40% of the control group (Fig. 6A). WB analysis also confirmed the protein level in A549 and NCI-H1299 cells (Fig. 6B). These results showed that REMAIN effectively suppressed the expression of ADAM9, a target gene of REMAIN. Indeed, REMAIN significantly inhibited cell migration and invasion compared with controls (Fig. 6C and D). Consistent with the uncovered mechanistic relation between miR-126-3p and ADAM9, the knockdown of ADAM9 repressed the migration and invasion capabilities of LUAD cells (Fig. 6C and D). To investigate the function of ADAM9 in the EMT progression and growth, we treated A549 and NCI-H1299 cells with REMAIN or si-ADAM9. First, REMAIN reduced growth and colony formation in A549 and NCI-H1299 cell lines (Figure S13A and B). Additionally, ADAM9 knockdown also inhibited both cell proliferation and colony formation of A549 and NCI-H1299 cells (Figure S13A and B). REMAIN, or si-ADAM9, increased the expression of E-cadherin and reduced levels of N-cadherin, Vimentin, and Snail (Fig. 6E). Hence, miR-126-3p/ADAM9 may be involved in controlling the EMT in LUAD, generally considered a critical mechanism in tumor cell metastasis.

### Biodistribution analysis

**Fig. 7:**
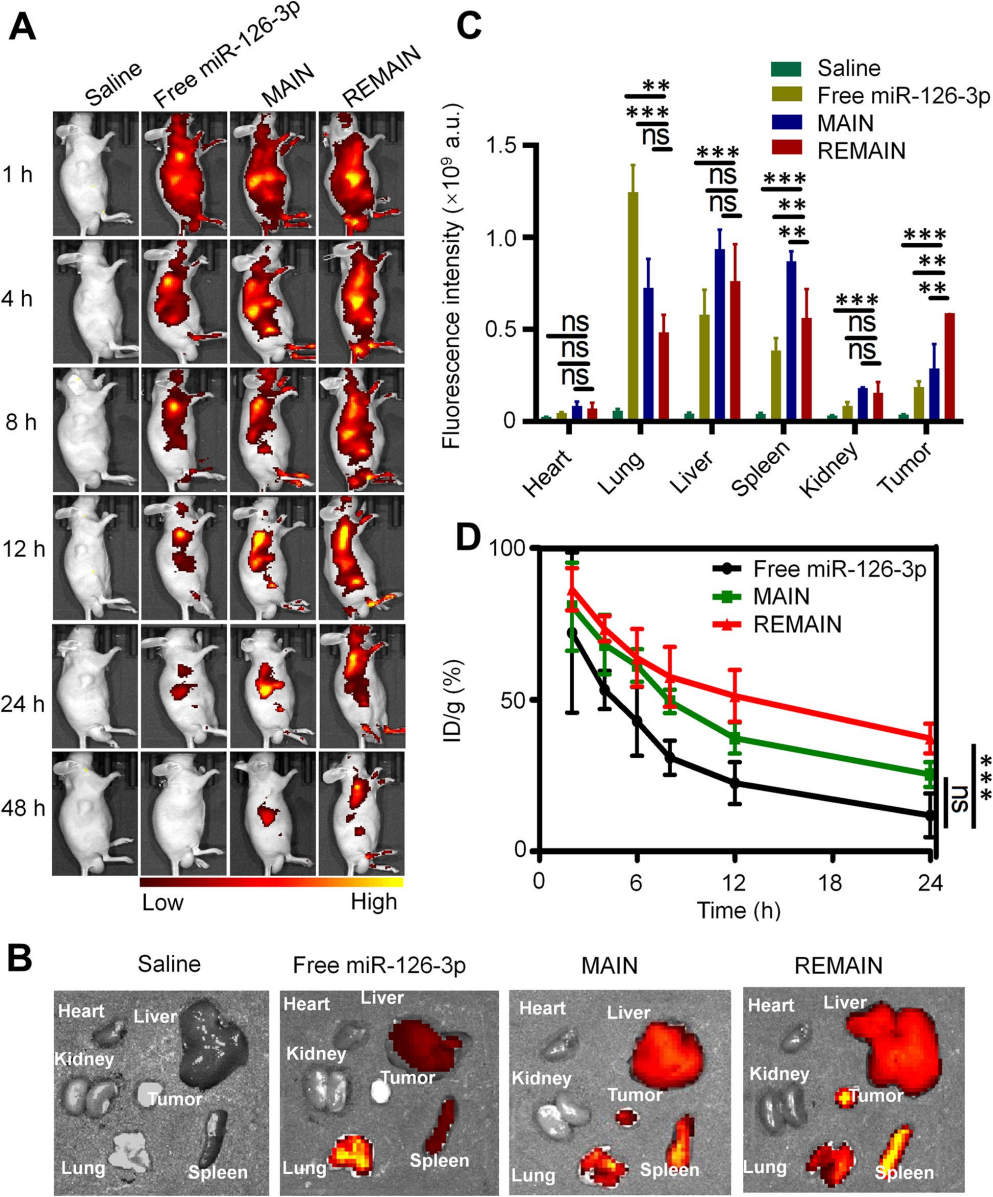
Biodistribution and circulation of different nanoparticles in vivo. **A** Biodistribution of nanoparticles in vivo. **B** Ex-vivo images of the major organs. **C** The quantitative analysis of the fluorescence in the major organs and tumors. **D** Circulation lifetime of nanoparticles. *, *P* < 0.05; **, *P* < 0.01; ***, *P* < 0.001

To monitor the distribution and tumor accumulation of REMAIN, we administrated REMAIN with Cy5-labeled miRNA to NCI-H1299 tumor-bearing mice. As shown in Fig. 7A, REMAIN was widely distributed throughout the whole body of mice within 4 h after tail vein injection. Higher signal intensity was observed in the REMAIN-treated groups than in free miR-126-3p or MAIN groups, while no fluorescent signal was detected in the saline-treated group. At 48 h after injection, the organs and tissues were isolated. The red fluorescence of REMAIN was much stronger compared with MAIN at the tumor sites, indicating that the RBCM coating promoted the accumulation and retention of miR-126-3p in tumors. Moreover, the *ex-vivo* imaging indicated that REMAIN showed the strongest fluorescence in the tumors than MAIN or free miR-126-3p (Fig. 7B and C), which may be due to REMAIN coated by RBCs escaping from phagocytosis by immune cells and increasing the circulation time [33]. The circulation lifetimes of REMAIN and MAIN were also measured. REMAIN showed a significantly increased circulation lifetime with over 70% present 6 h after administration. Notably, over 40% of REMAIN (ID/g) was available after 24 h (Fig. 7D). The prolong circulation lifetime is attributed to the structure of RBCM, which is composed of a mixture of lipids, proteins, and carbohydrates. The lipids mainly contribute to the bilayer structure and fluidity of RBCM. The membrane proteins such as transmembrane or membrane-anchored ones, as well as carbohydrates endow the membranes with versatile functionalities, such as recognition, specificity, or immunomodulation [19]. Interestingly, the camouflage with cell membranes exhibited right-side-out membrane orientation [34, 35], which displays the immunomodulatory markers such as the “self-marker” CD47 on the surface with the same density as the original RBCs [36, 37]. Previous work also indicated that the surface modifications with RBC membranes improved the circulation lifetime of nanoparticles [38]. These results suggested that the longer circulation lifetime of red cell membrane coating miR-126-3p-containing nanoparticles facilitated the accumulation in tumors via enhanced permeability and retention and improved the therapeutic effects.

### Antitumor effects of the nanoparticles in vivo

**Fig. 8:**
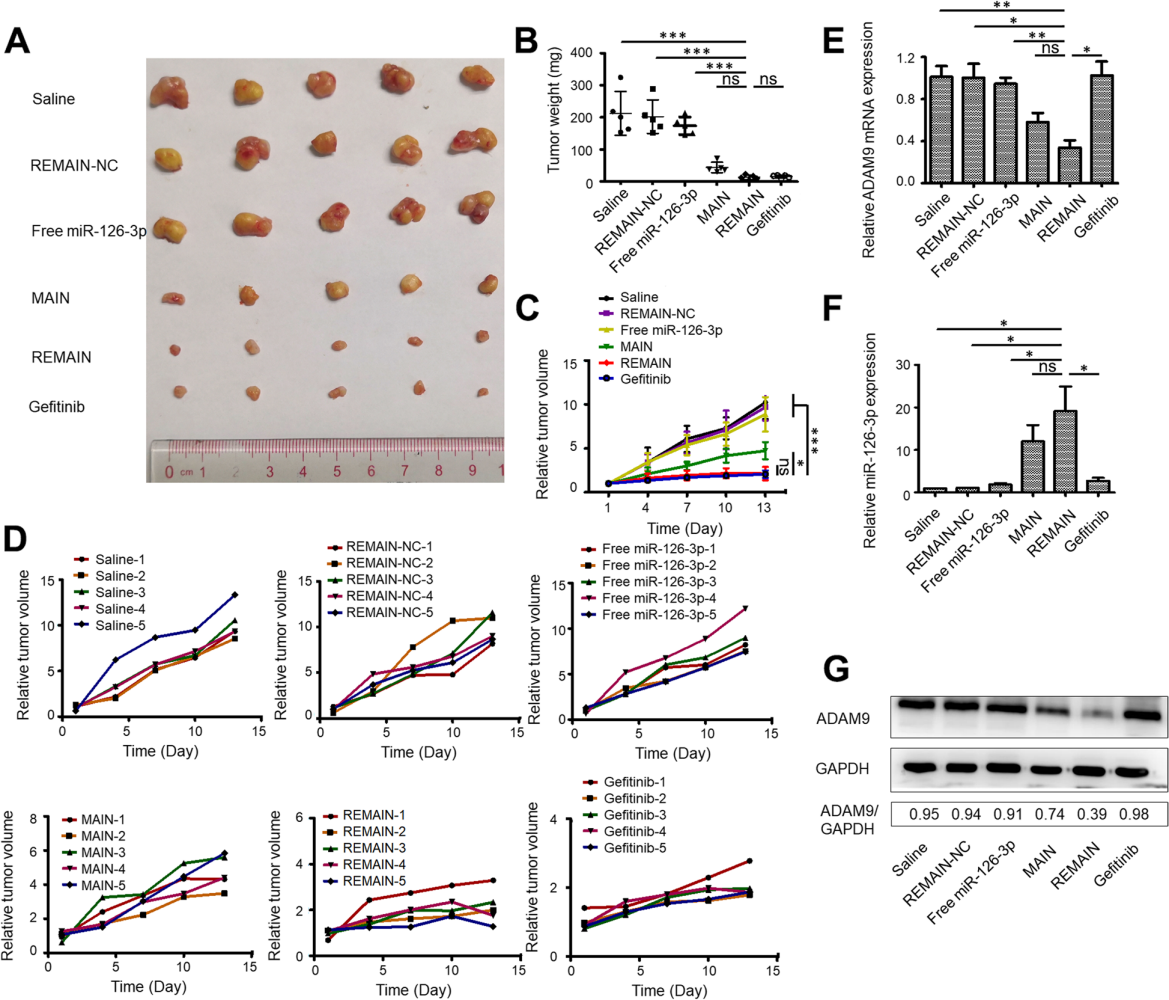
The effect of different formulations of nanoparticles on tumor growth in vivo (*n* = 6). **A** The excised tumors were imaged. **B** The excised tumors were weighed. **C** and **D** The changes in the tumor volumes were determined. The mRNA levels of ADAM9 (**E**) and miR-126-3p (**F**) were detected in tumors. **G** The protein levels of ADAM9 were measured in tumors. *, *P* < 0.05; **, *P* < 0.01; ***, *P* < 0.001

To evaluate the antitumor effects of REMAIN in vivo, we evaluated the tumor inhibition using a xenograft nude mouse model. The formulations were injected through the tail vein. Saline, REMAIN-NC, and free miR-126-3p treated mice were used as controls. As shown in Fig. 8A-D, we observed no significant difference in tumor size, weight, and volume among mice treated with saline, REMAIN-NC, or free miR-126-3p. However, the treatment with REMAIN, MAIN, or Gefitinib significantly inhibited tumor growth, as indicated by the tumor size, weight, and volume of extracted tumors. Compared to the MAIN group, the REMAIN group exhibited a higher antitumor effect, similar to the Gefitinib-treated group. REMAIN and MAIN showed no toxic effects, as the bodyweight of REMAIN and MAIN-treated mice showed no significant difference from the saline-treated group (Figure S14). Furthermore, RT-qPCR analysis demonstrated that REMAIN significantly suppressed ADAM9 expression and increased miR-126-3p levels (Fig. 8E and F). WB analysis also indicated that ADAM9 protein levels were reduced in the REMAIN-treated group (Fig. 8G). Moreover, hematoxylin–eosin (HE) staining demonstrated that none of the treatments induced obvious histological changes in major organs, indicating no significant toxicity to mice (Figure S15). Moreover, the apoptosis of tumor tissues was evaluated using TUNEL staining. Our results showed that REMAIN, MAIN, and Gefitinib had a higher apoptosis rate than the saline, REMAIN-NC, and free miR-126-3p-treated mice (Figure S16). These results indicated that REMAIN efficiently suppressed tumor growth by delivering miR-126-3p to the tumor tissues in the tumor-bearing models.

## Discussion

Although miRNAs in lung cancer have been studied for years, they have not entered the clinical application stage so far. One of the biggest challenges is identifying the best miRNA candidates and/or targets for lung cancer treatment. To ensure the reliability of our results, the upregulated or downregulated miRNAs with more than five-fold change were included in our research for further study. Based on our microarray assay, the promoter methylation-associated expression of miR-126-3p was identified as one of the most downregulated miRNAs among the differentially regulated miRNAs in LUAD tissues [39]. In addition, the development of miRNA-based therapeutics is limited by miRNA delivery vehicles that possess higher transfection efficiency. To enhance the efficacy of miRNA delivery, we developed an RBCM decorated nanoparticle to deliver therapeutic miRNA for LUAD therapy. A key point of our research is that we first focused on the mechanical characterization and practical application of miR-126-3p in LUAD.

The dysregulation of miRNA happens in various cancers, associated with genetic and epigenetic alterations [40, 41]. The downregulation of miR-126-3p has been revealed in malignant lung tumors. These studies implied that miR-126-3p might be a predictive biomarker for lung cancer diagnosis and treatment. However, the clinical significance of promoter methylation-associated expression of miR-126-3p still needs further study. We investigated the role of miR-126-3p dysregulation in lung cancer development. First, downregulation of miR-126-3p in LUAD cells resulted from methylation status, and the opposite trend was observed between its expression and the promoter methylation of miR-126-3p. Furthermore, miR-126-3p methylation was significantly associated with tumor metastasis. The promoter methylation-associated downregulation of miR-126-3p contributed to lung cancer metastasis, highlighting that either the expression or methylation status of miR-126-3p might act as prognostic/predictive markers in LUAD.

Based on our current results, miR-126-3p possesses the potential as an independent prognostic factor for LUAD. To enhance the ability of miR-126-3p to diffuse into cells, we prepared miR-126-3p-loaded nanoparticles, REMAIN, for lung cancer therapy. REMAIN exhibited a series of advantages: (1) The RBC membrane camouflage prolonged the circulation time of REMAIN; (2) REMAIN showed an MMP2-stimulated release effect, improving the miR-126-3p release in the LUAD cells; and (3) The biomimetic nanoparticles showed high safety in vivo. Significantly, the enhanced circulation lifetime for REMAIN was interesting. RBCM is composed of a mixture of lipids, proteins, and carbohydrates. The lipids mainly contribute to the bilayer structure and fluidity of RBCM. The membrane proteins such as transmembrane or membrane-anchored ones, as well as carbohydrates endow the membranes with versatile functionalities, such as recognition, specificity, or immunomodulation [19]. Interestingly, the cell-membrane-coated nanoparticles exhibited right-side-out membrane orientation [34, 35], which displays the immunomodulatory markers such as the “self-marker” CD47 on the surface with the same density as the original RBCs [36, 37]. Previous work also indicated that the surface modifications with RBC membranes improved the circulation lifetime of nanoparticles [38].

Consistent with previous studies, we confirmed ADAM9 as a direct target of miR-126-3p and found a reverse association between miR-126-3p and ADAM9 mRNA levels in LUAD patients. Our present study also revealed that nanoparticles carrying miR-126-3p targeted ADAM9 directly, and REMAIN resulted in ADAM9 downregulation. Moreover, ADAM9 expression was inhibited by 5-Aza-dC. In vitro studies also confirmed that nanoparticles carrying miR-126-3p or ADAM9 knockdown inhibited tumor growth and metastasis by regulating cell proliferation, colony formation, migration, invasion, and EMT. Our findings indicate that the stealth and MMP2-stimulated biomimetic nanoparticles carrying miR-126-3p could inhibit LUAD metastasis by targeting ADAM9.

## Conclusion

In conclusion, our work demonstrated that miR-126-3p is an effective repressor for LUAD inhibition, targeting to ADAM9 in LUAD. We developed a potent and effective nanosystem based on RBCM-coated MMP2-stimulated peptide-binding miR-126-3p. This novel style of biomimetic nanoparticles enabled miR-126-3p to effectively induce cancer cell apoptosis and inhibit tumor growth and metastasis by targeting ADAM9. Our work highlights a novel miRNA and its underlying mechanism in LUAD inhibition in the clinical samples. Furthermore, we developed a novel style of biomimetic nanoparticles for miRNA delivery in the treatment of LUAD.

## Supplementary Information

**Additional file 1.**

## Data Availability

The data supporting the findings of this study are available within the article and its supplementary information files and from the corresponding author upon request.

## Abbreviations

LUAD: Lung adenocarcinoma
MMP2: Matrix metalloproteinase-2
PLGLAG: Pro-Leu-Gly-Leu-Ala-Gly
RBCM: Red blood cell membrane
MAIN: MMP2 stimulated peptide/miRNA-126-3p
REMAIN: Red blood cell membrane/MMP2 stimulated peptide/miRNA-126
CCK-8: Cell counting kit-8
NC: Negative control
WT: Wild-type
MT: Mutant
ADAM9: A disintegrin and a metalloprotease 9
qRT-PCR: Quantitative real-time PCR
MSP: Methylation**-**specific PCR
H&E: Hematoxylin and eosin
5-Aza-dC: 5-Aza-2’-deoxycytidine
UTR: Untranslated region
EMT: Epithelial- mesenchymal transition
miRNAs: MicroRNAs
