# Supplementary material for: The reversion of DNA methylation-induced miRNA silence via biomimetic nanoparticles-mediated gene delivery for efficient lung adenocarcinoma therapy
Source: Mol Cancer. 2022 Sep 28;21:186. doi: 10.1186/s12943-022-01651-4 (PMC9516831; doi:10.1186/s12943-022-01651-4)
Supplement: Supplementary file 1 — Additional file 1. [file 12943_2022_1651_MOESM1_ESM.docx]

**Supplemental data**

**
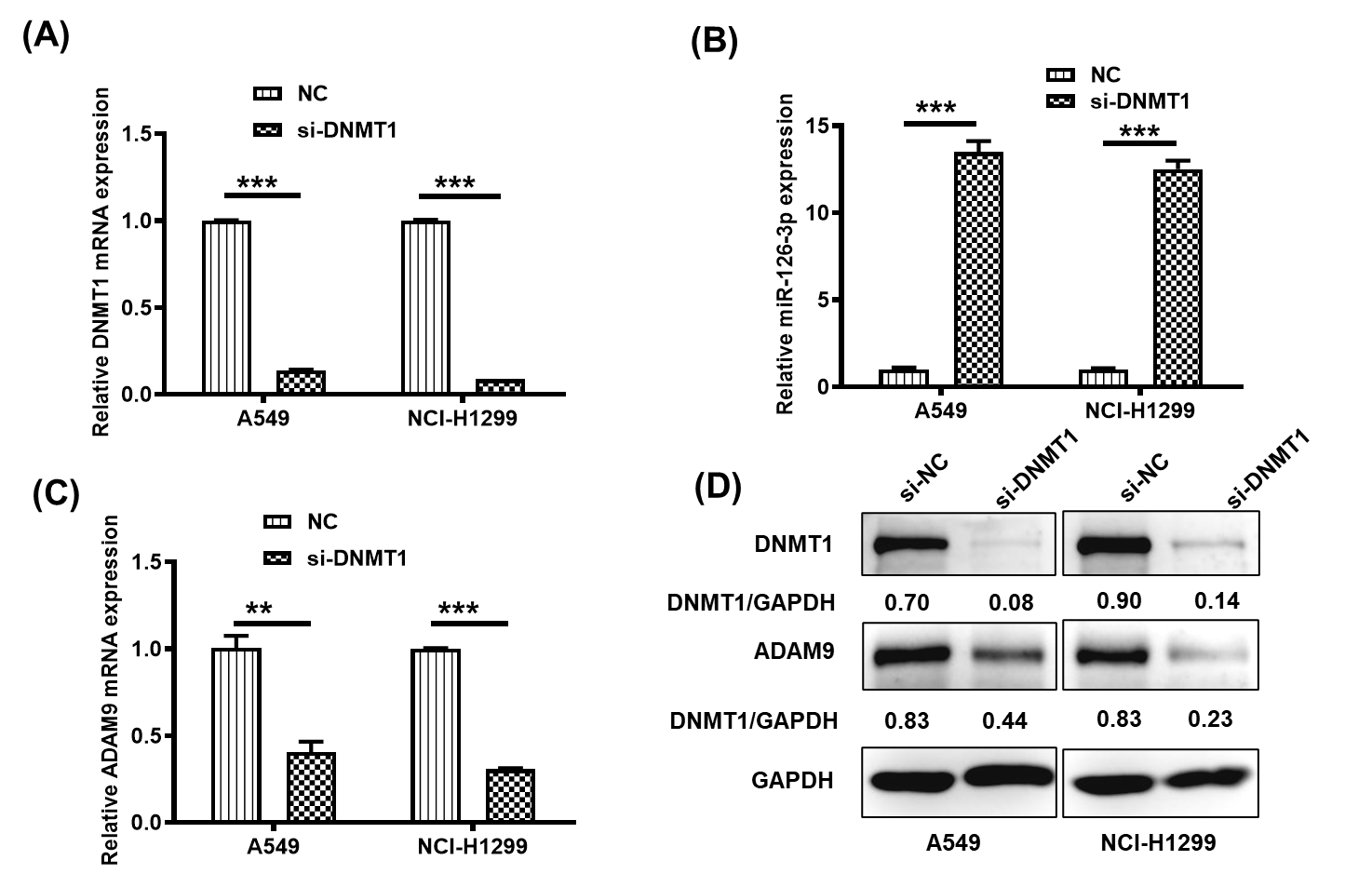
**

**Figure S1. The effect of silencing DNMT1 on miR-126-3p and ADAM9 expression.**

(A) The mRNA levels of DNMT1 were measured in si-DNMT1-treated A549 and NCI-H1299 cells 48 hours post-transfection, respectively (n = 3). (B) The expression levels of miR-126-3p were measured in si-DNMT1-treated A549 and NCI-H1299 cells 48 hours post-transfection, respectively (n = 3). (C) The mRNA levels of ADAM9 were measured in si-DNMT1-treated A549 and NCI-H1299 cells 48 hours post-transfection, respectively (n = 3). (D) The protein levels of ADAM9 and DNMT1 were measured in si-DNMT1-treated A549 and NCI-H1299 cells 48 hours post-transfection, respectively (n = 3).


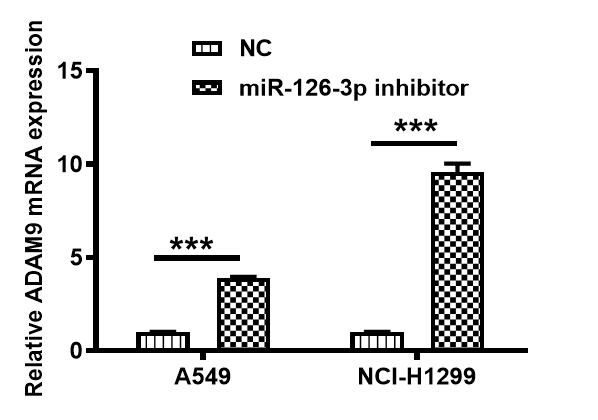


**Figure S2. The effect of miR-126-3p inhibition on ADAM9 expression.** NCI-H1299 cells were transfected with miR-126-3p inhibitor (150 nM) using Lipo3000. After 48 h, the expression levels of ADAM9 were evaluated by RT-qPCR assay.

**
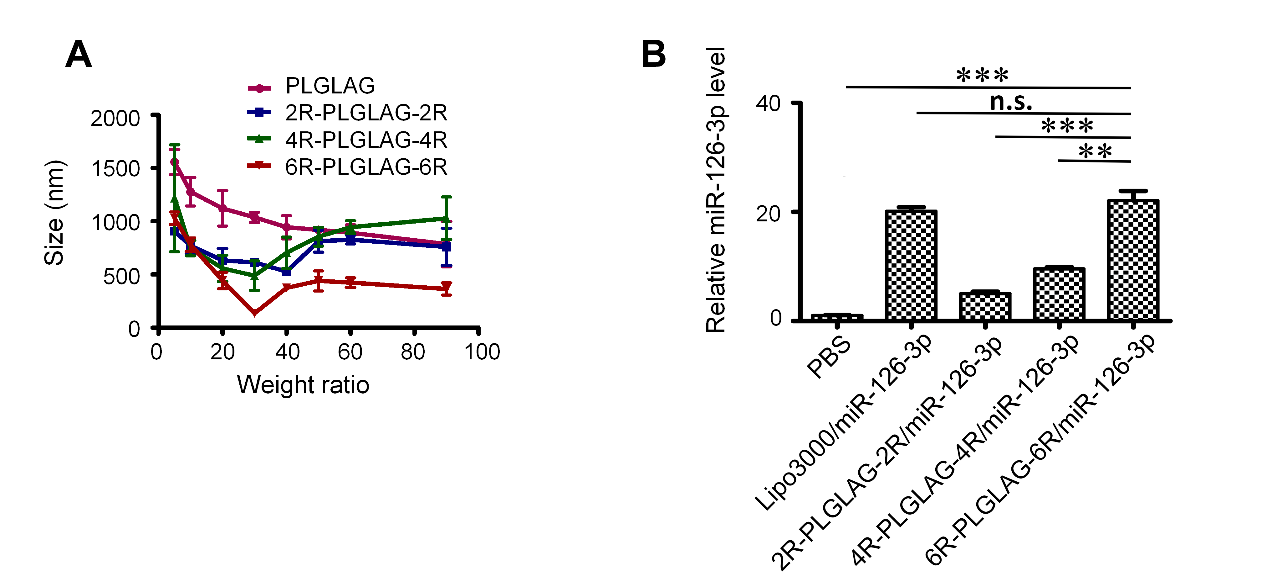
**

**Figure S3. The effect of different amounts of arginine on the formation of nanoparticles.** (A) The sizes of 2R-PLGLAG-2R/miR-126-3p, 4R-PLGLAG-4R/miR-126-3p, and 6R-PLGLAG-6R/miR-126-3p. (B) miR-126-3p levels were analyzed in NCI-H1299 cells after treatment with Lipo3000/miR-126-3p, 2R-PLGLAG-2R/miR-126-3p, 4R-PLGLAG-4R/miR-126-3p, or 6R-PLGLAG-6R/miR-126-3p, respectively. *, *P* < 0.05; **, *P* < 0.01; ***, *P* < 0.001.


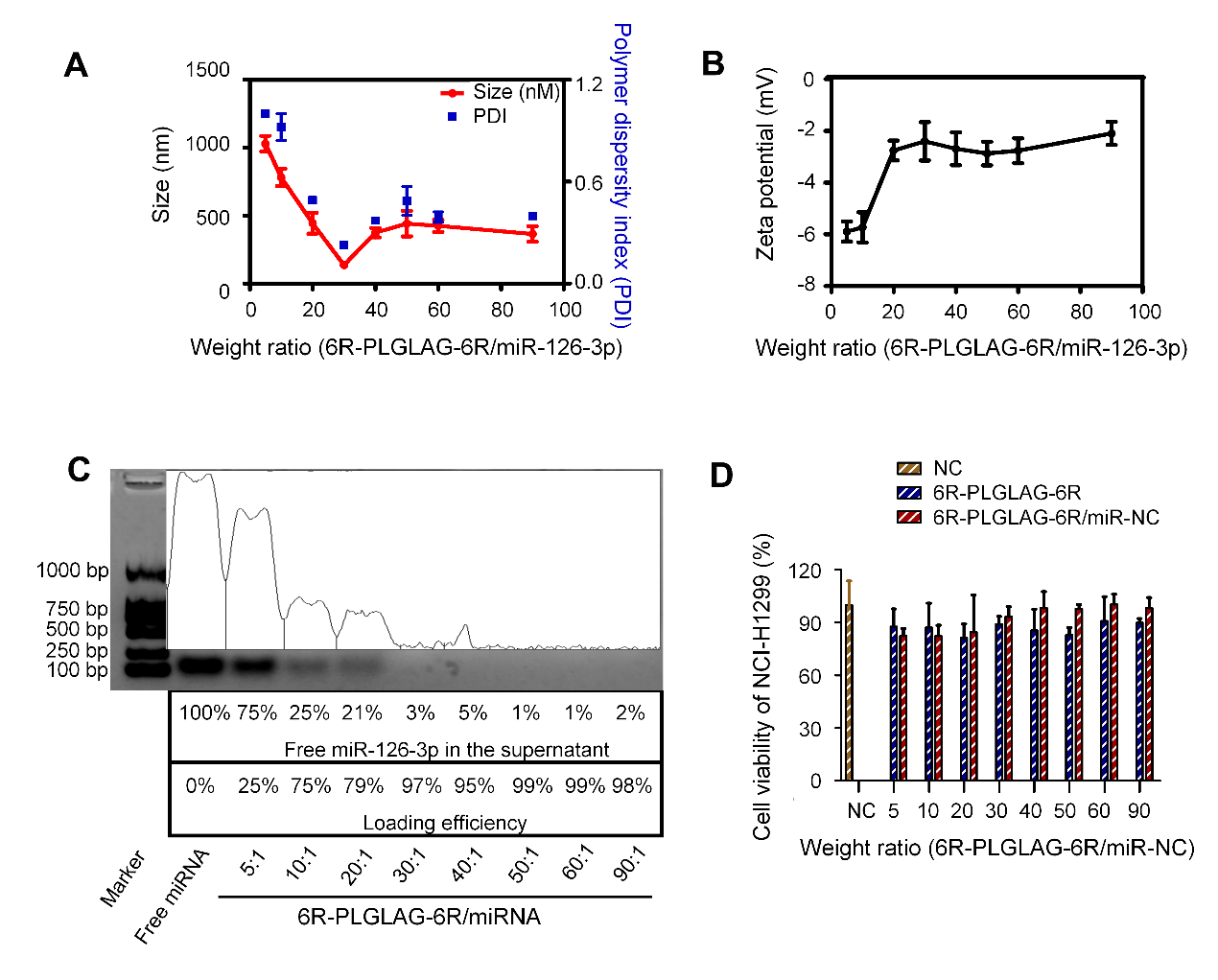


**Figure S4.** **The sizes and Zeta potentials of MAIN.** (A) The sizes and (B) Zeta potentials of 6R-PLGLAG-6R/miR-126-3p. The combination of 6R-PLGLAG-6R and miR-126-3p in different weight ratios (5, 10, 20, 30, 40, 50, 60, and 90). (C) The loading efficiency of 6R-PLGLAG-6R with miR-126-3p. (D) Cell viability of 6R-PLGLAG-6R/miR-NC was analyzed by CCK-8 assay.


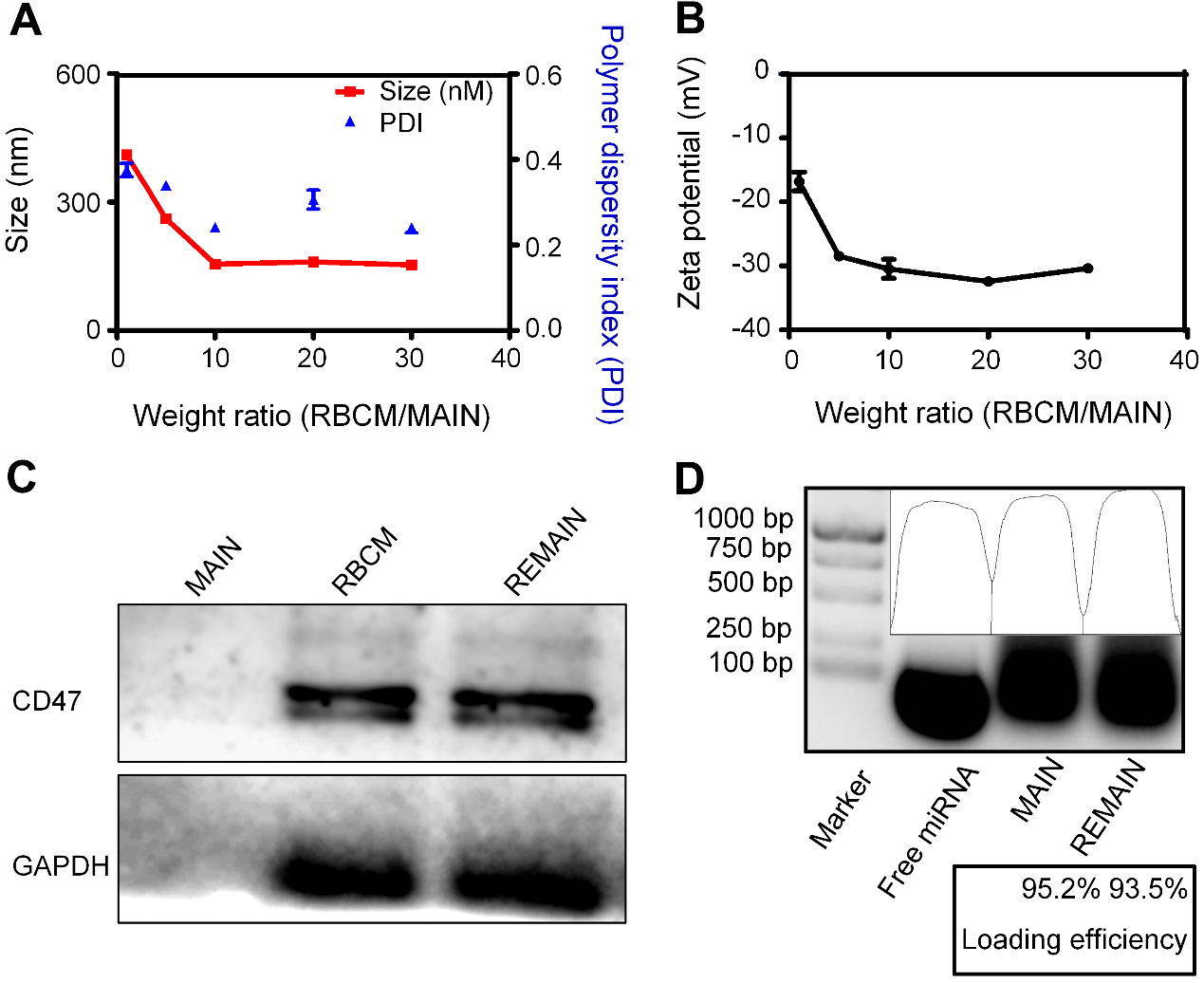


**Figure S5.** **The sizes and Zeta potentials of MAIN and REMAIN.**

(A) The sizes and (B) Zeta potentials of RBCM/MAIN. The combination of RBCM and MAIN in different weight ratios (1, 5, 10, 20 and 30). (C) WB analysis of CD47 level. (D) The loading efficiency of the nanoparticles. Before the agarose gel electrophoresis, free miR-126-3p, MAIN, and REMAIN was incubated with 4 mg/mL heparin for 2 h, respectively.


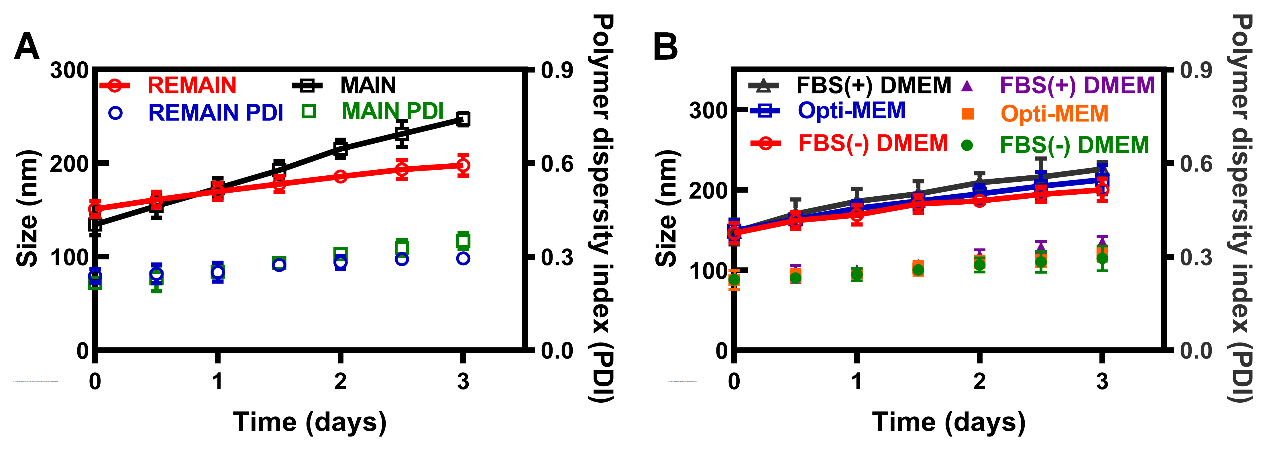


**Figure S6. The stability of different nanoparticles.** (A) Size and PDI of MAIN or REMAIN in PBS. (B) Size and PDI of REMAIN in the medium with different concentration of FBS.


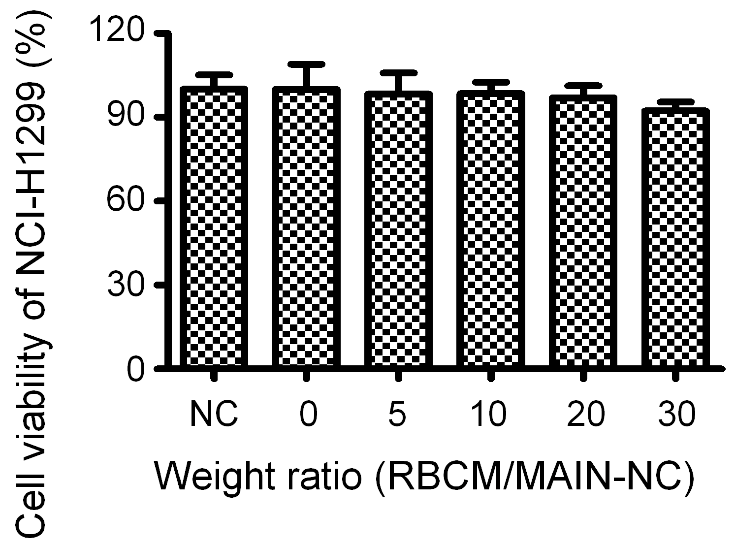


**Figure S7. Cell viability of RBCM/MAIN -NC was analyzed by CCK-8 assay.**


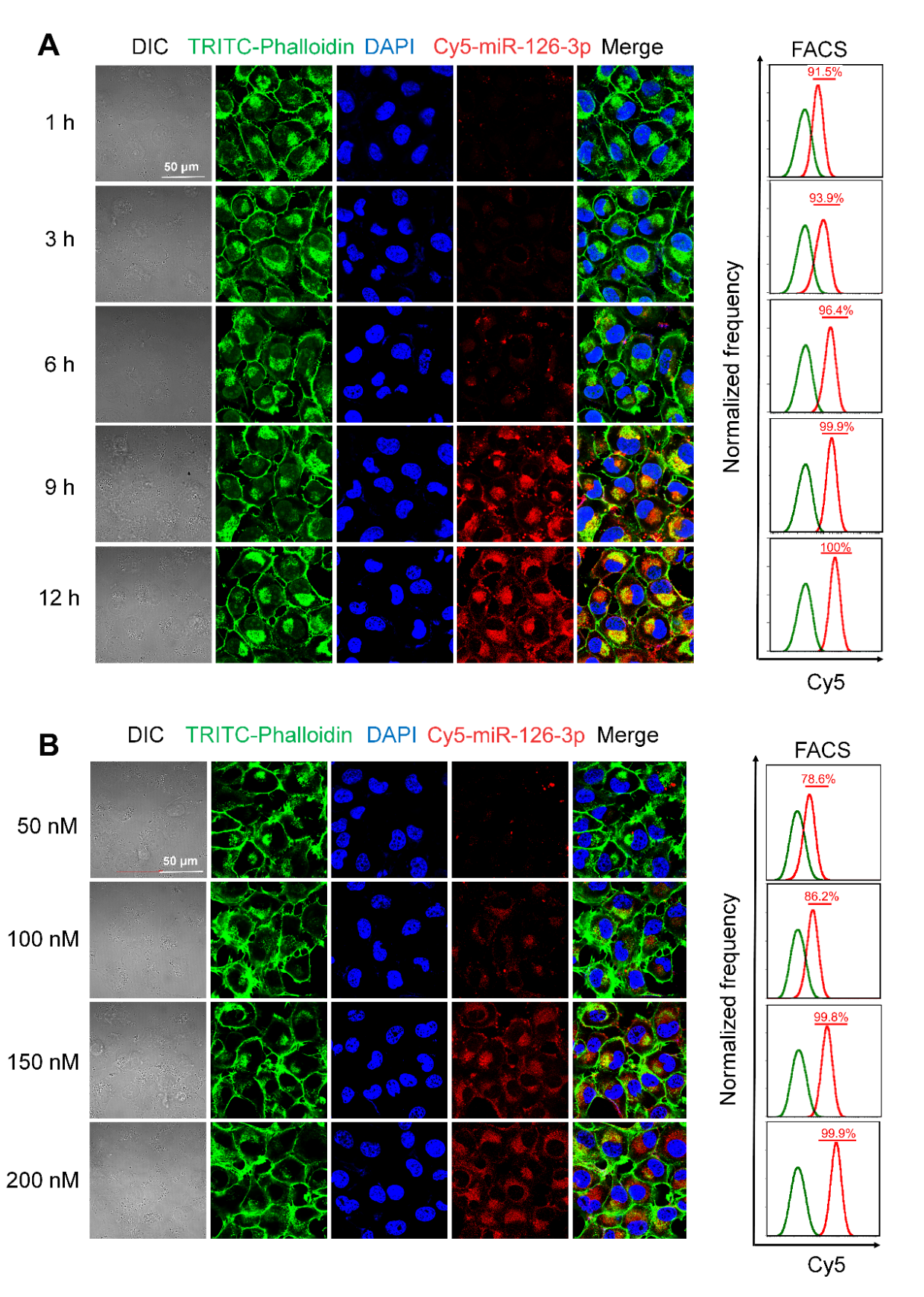


**Figure S8. The evaluation of cellular uptake** **in a time- and dose-dependent manner.** (A) The cellular uptake of REMAIN in a time-dependent manner. NCI-H1299 cells were transfected with REMAIN (Cy5-miR-126-3p equivalent to 150 nM) for 1, 3, 6, 9 and 12 h, respectively. (B) The cellular uptake of REMAIN in a dose-dependent manner. NCI-H1299 cells were transfected with various concentrations of REMAIN (Cy5-miR-126-3p equivalent to 50, 100, 150, and 200 nM) for 9 h, respectively.


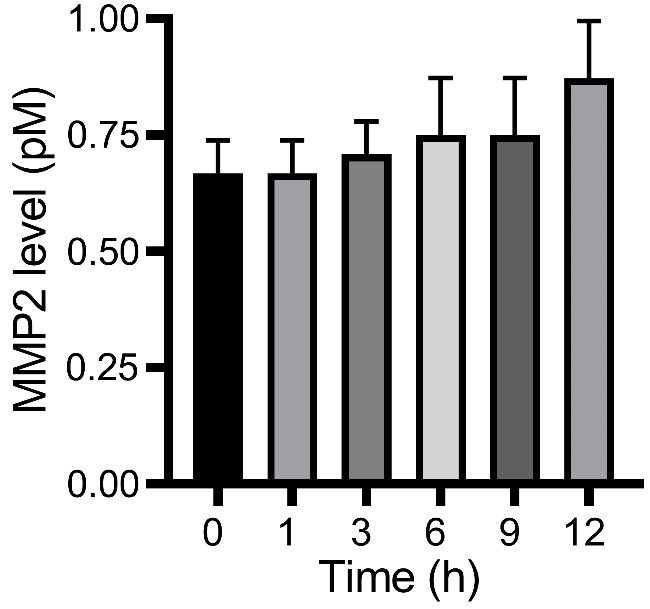


**Figure S9. The MMP2 level in the culture medium.** The culture medium was obtained at the time points of 0, 1, 3, 6, 9, and 12 h, respectively. To analyze quantitatively the MMP2 level, the culture medium was analyzed by MMP2 ELISA Kit.


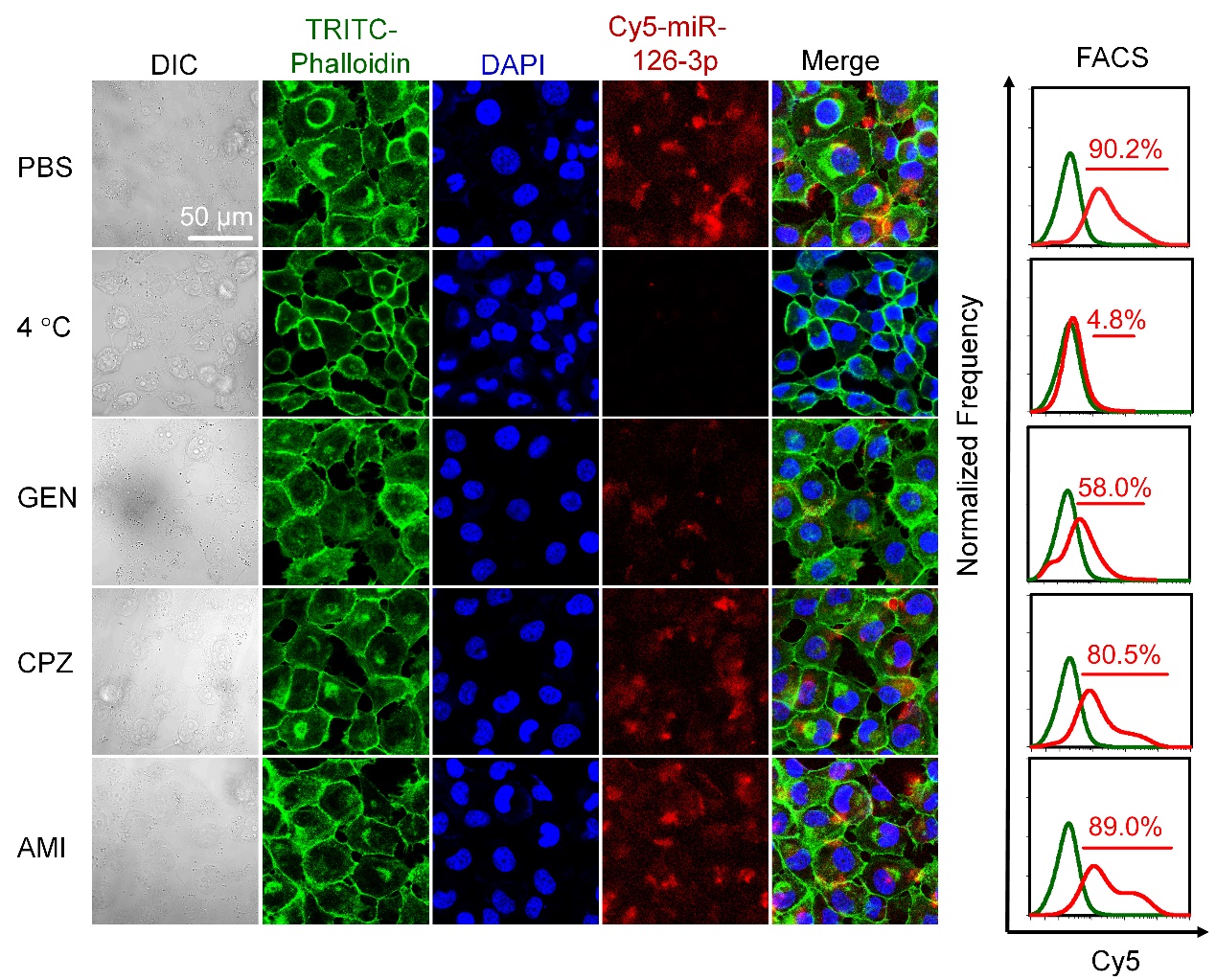


**Figure S10. CLSM analysis of the endocytosis mechanism.** The cells were treated with PBS, 4 °C, GEN, CPZ, or AMI for 2 h, respectively. And then the cells treated with REMAIN for 9 h. After 9 h, the cells were analyzed with CLSM and FACS.


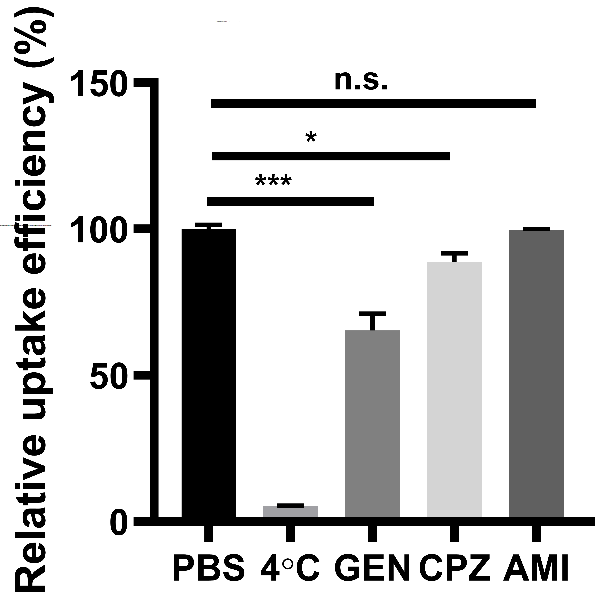


**Figure S11. FACS analysis of the endocytosis mechanism.** The cells were treated with PBS, 4 °C, GEN, CPZ, and AMI, respectively.


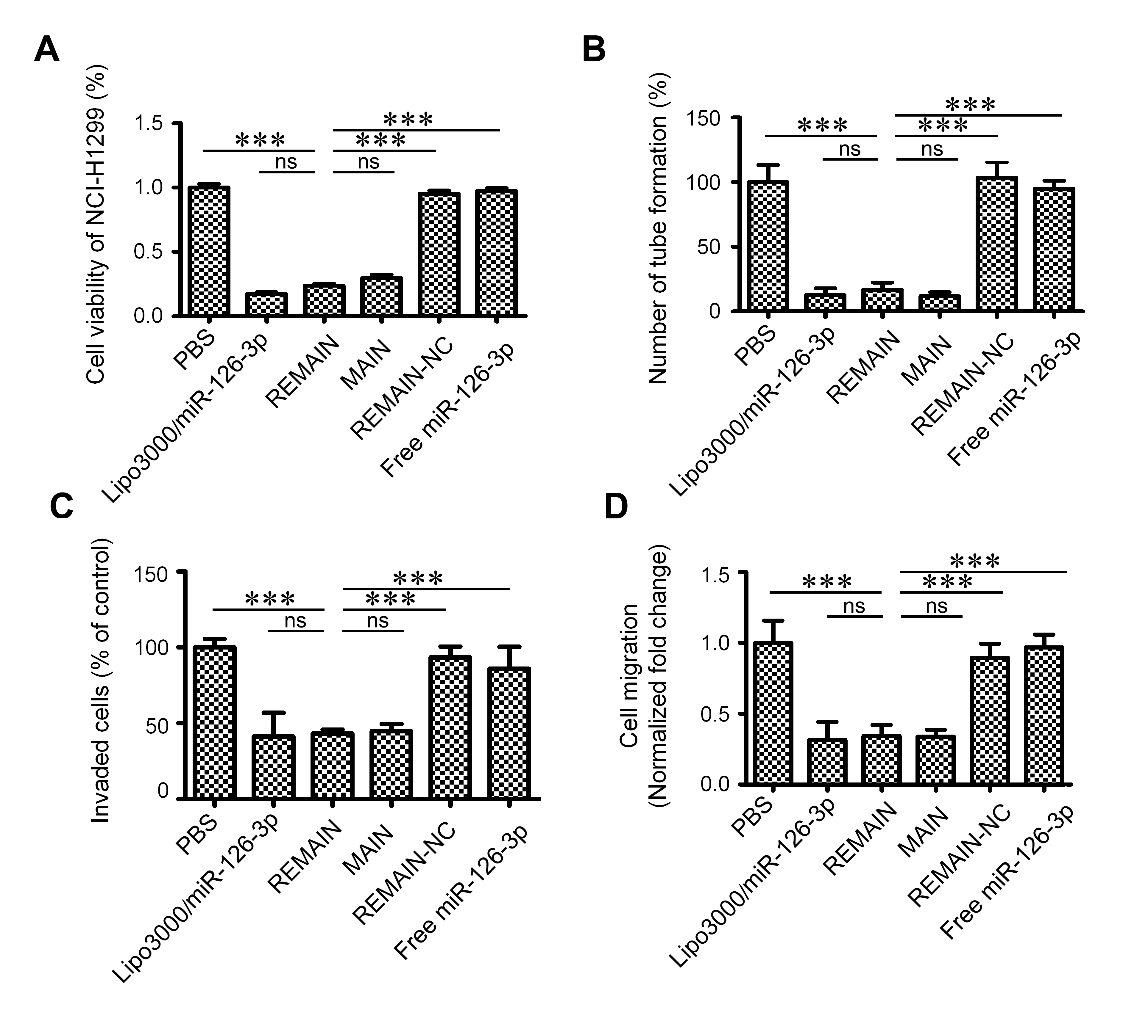


**Figure S12. The effect of different formulations of nanoparticles on NCI-H1299 cells *in vitro*.** (A) Cell viability, (B) Tube formation of HUVEC, (C) Cell invasion and (D) Cell migration and were quantitatively analyzed after treatment with PBS, Lipo3000/miR-126-3p, REMAIN, MAIN, REMAIN-NC or free miR-126-3p, respectively. *, *P* < 0.05; **, *P* < 0.01; ***, *P* < 0.001.

**
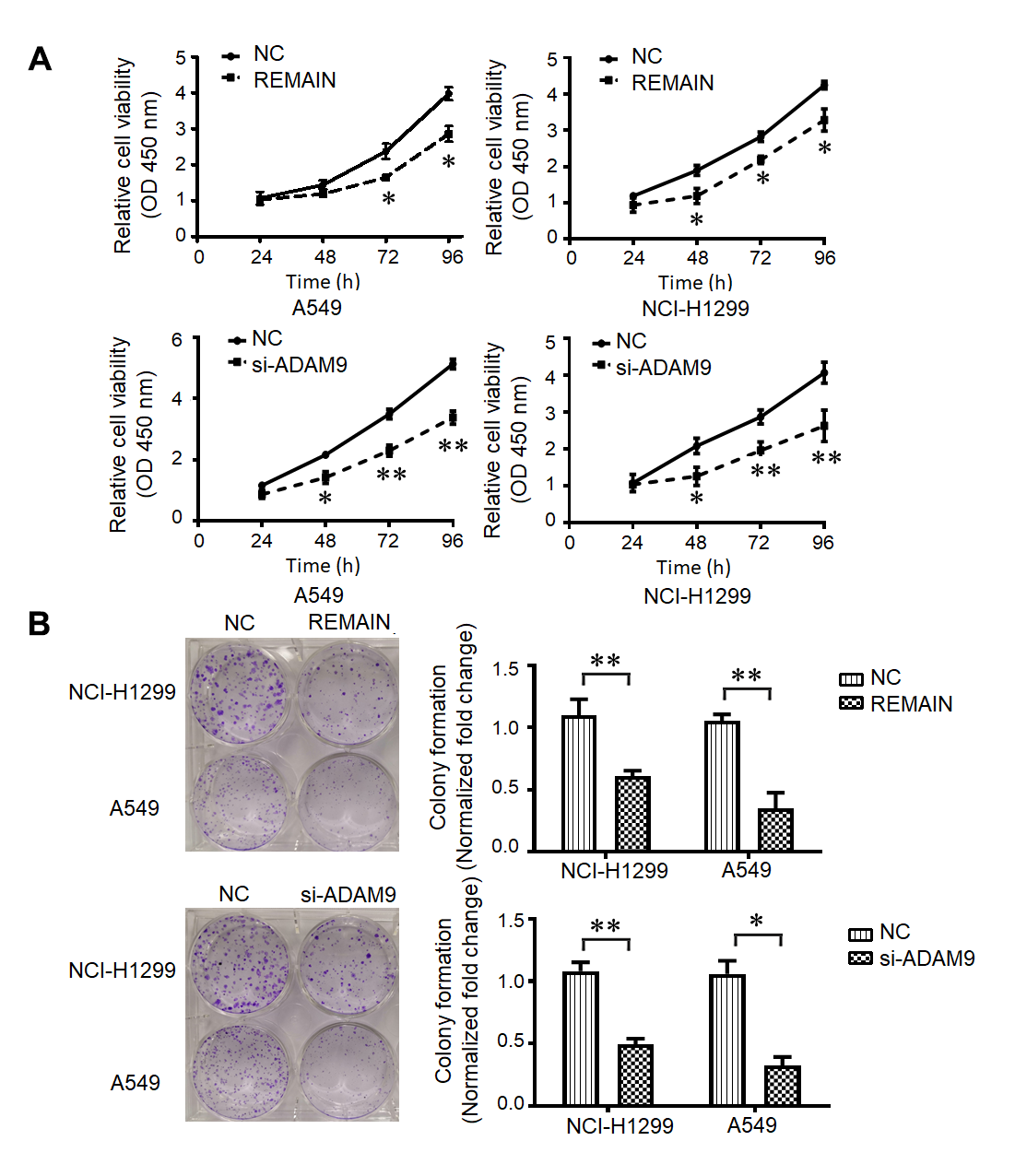
**

**Figure S13. REMAIN or ADAM9 knockdown inhibits proliferation and colony formation of LUAD cells.** A549 and NCI-H1299 cells were treated with REMAIN or si-ADAM9 for 48 h. Cell proliferation (A) and colony formation (B) were analyzed after indicated treatments. Representative images and data from three independent measures. *, *P* < 0.05; **, *P* < 0.01; ***, *P* < 0.001.


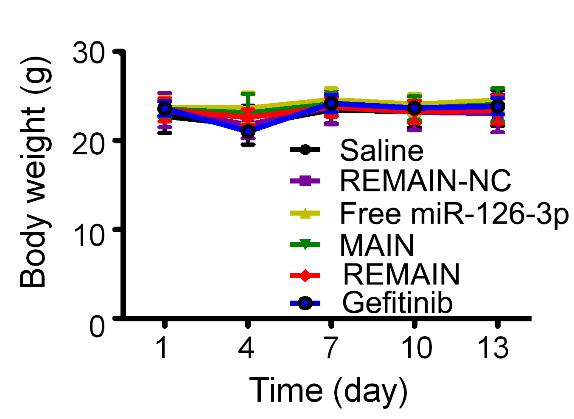


**Figure S14. The changes of the body weight (n=6).**


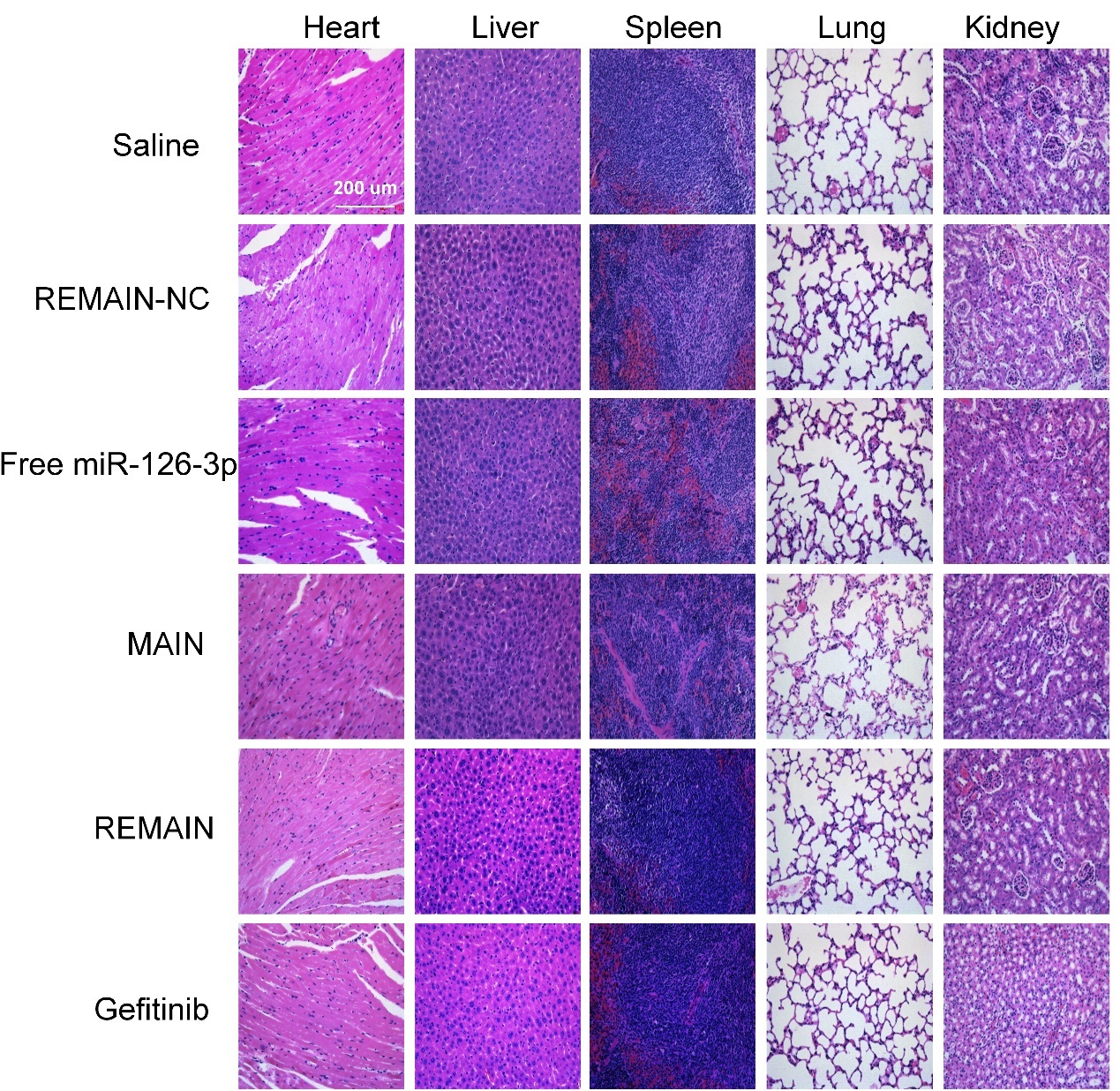


**Figure S15.** **Representative H&E-stained slices of major organs.**


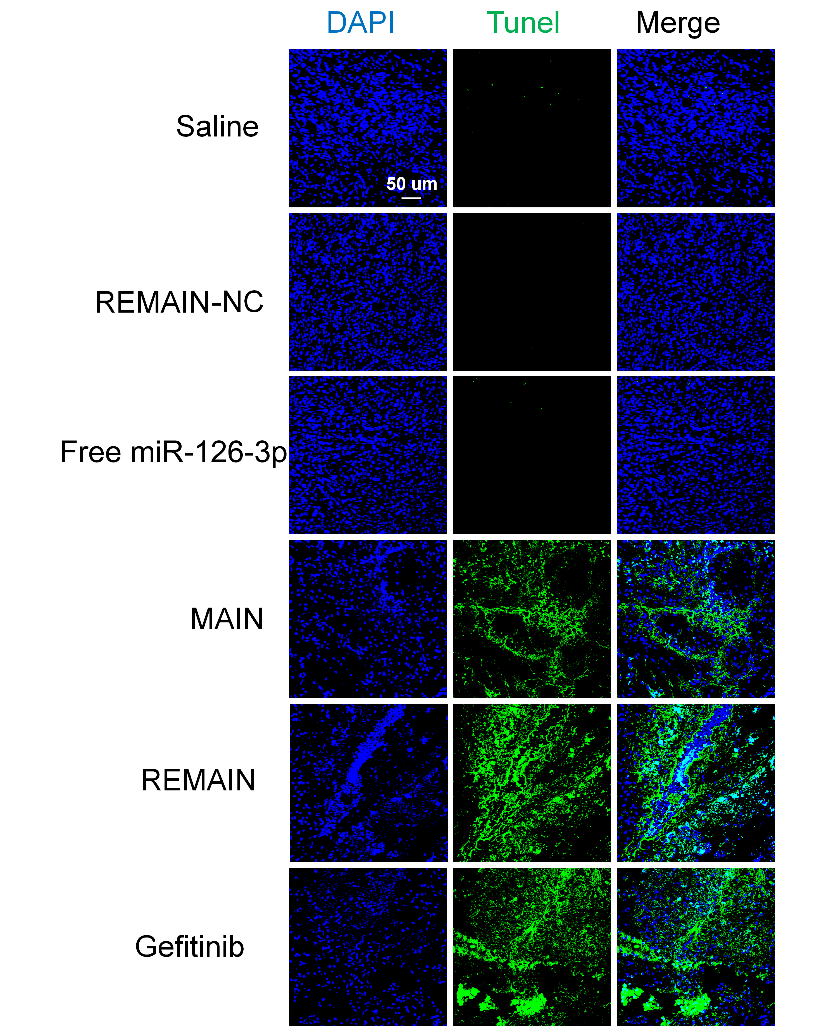


**Figure S16.** **TUNEL staining in the tumor tissues.**

**Table S1. Primers for RT-qPCR**

| Gene | Sequence (5'‑3') |
| --- | --- |
| miRNA‑126-3p Forward | GCTGGCGACGGGACATTA |
| miRNA‑126-3p Reverse | CGCATTATATACTCACGGAAGG |
| U6 Forward | GCAGGGGCCATGCTAATCTTCTCTGTATCG |
| U6 Reverse | CAAACAAAAAAACTAAAACCCCACAACAAACA |
| ADAM9 Forward | CTACAACGGCATGTGCCAG |
| ADAM9 Reverse | CACTGAAGCTTTCCACACAAA |
| GAPDH Forward | CCCTCAACGACCACTTTGTC |
| GAPDH Reverse  miRNA‑126-3p NC  miRNA‑126-3p mimic  miRNA‑126-3p inhibitor | AGGGGAGATTCAGTGTGGTG  CAGUACUUUUGUGUAGUACAA  UCGUACCGUGAGUAAUAAUGCG  CGCAUUAUUACUCACGGUACGA |

**Table S2 MSP primers for miR-126-3p**

| Gene | Sequence (5'‑3') |
| --- | --- |
| miRNA‑126-3p-U Forward | TTGGCGGTCGGGTTTGGTC |
| miRNA‑126-3p-U Reverse | TAAAACCCCGCGACGAACG |
| miRNA‑126-3p-M Forward | TTGGTTTTTGGTGGTTGGGTTTGGTT |
| miRNA‑126-3p-M Reverse | CAAACAAAAAAACTAAAACCCCACAACAAACA |

**Table S3. The relationship between miR-126-3p methylation and clinicopathologic characteristics of patients with LUAD**

| Clinicopathologic characteristics | Total cases (n, %) | Cases with miR-126-3p methylation (n, %) | *P* value |
| --- | --- | --- | --- |
| Age |  |  | 0.1051 |
| ≤60 | 42 (62.7) | 27 (64.3) |  |
| >60 | 25 (37.3) | 11 (44.0) |  |
| Sex |  |  | 0.4398 |
| Female | 29 (43.3) | 18 (62.1) |  |
| Male | 38 (56.7) | 20 (52.6) |  |
| Degree of differentiation |  |  | 0.0465* |
| Well and moderately | 37 (55.2) | 25 (67.6) |  |
| Poorly | 30 (44.8) | 13 (43.3) |  |
| Lymph node metastasis |  |  | 0.0022** |
| Yes | 28 (41.8) | 22 (78.6) |  |
| No | 39 (58.2) | 16 (41.0) |  |
